# The Influence of Minor Aortic Branches in Patient-Specific Flow Simulations of Type-B Aortic Dissection

**DOI:** 10.1007/s10439-023-03175-4

**Published:** 2023-03-26

**Authors:** C. Stokes, F. Haupt, D. Becker, V. Muthurangu, H. von Tengg-Kobligk, S. Balabani, V. Díaz-Zuccarini

**Affiliations:** 1 Department of Mechanical Engineering, University College London, London, UK; 2 Wellcome-EPSRC Centre for Interventional Surgical Sciences, University College London, London, UK; 3 Department of Diagnostic, Interventional and Pediatric Radiology, Inselspital, University of Bern, Bern, Switzerland; 4 Clinic of Vascular Surgery, Inselspital, University of Bern, Bern, Switzerland; 5 Centre for Translational Cardiovascular Imaging, University College London, London, UK

**Keywords:** Computational fluid dynamics, Aortic dissection, Intercostal arteries, Segmental arteries, Lumbar arteries, Inferior mesenteric artery, Patient-specific, 4D-flow MRI

## Abstract

Type-B aortic dissection (TBAD) is a disease in which a tear develops in the intimal layer of the descending aorta forming a true lumen and false lumen (FL). Because disease outcomes are thought to be influenced by haemodynamic quantities such as pressure and wall shear stress (WSS), their analysis *via* numerical simulations may provide valuable clinical insights. Major aortic branches are routinely included in simulations but minor branches are virtually always neglected, despite being implicated in TBAD progression and the development of complications. As minor branches are estimated to carry about 7–21% of cardiac output, neglecting them may affect simulation accuracy. We present the first simulation of TBAD with all pairs of intercostal, subcostal and lumbar arteries, using 4D-flow MRI (4DMR) to inform patient-specific boundary conditions. Compared to an equivalent case without minor branches, their inclusion improved agreement with 4DMR velocities, reduced time-averaged WSS (TAWSS) and transmural pressure and elevated oscillatory shear in regions where FL dilatation and calcification were observed *in vivo*. Minor branch inclusion resulted in differences of 60-75% in these metrics of potential clinical relevance, indicating a need to account for minor branch flow loss if simulation accuracy is sought.

**Supplementary Information:**

The online version of this article contains supplementary material which is available https://doi.org/10.1007/s10439-023-03175-4.

## Introduction

Aortic dissection (AD) is a life-threatening condition in which a tear forms in the innermost layer of the aorta, separating the true lumen (TL) from a false lumen (FL) as blood flows between the layers of the aortic wall. Type-B AD (TBAD) is characterised by a primary entry tear (PET) in the descending aorta. Uncomplicated cases of TBAD can be treated medically, but the disease is dynamic in nature; FL dilatation^15^ is a primary independent risk factor for long-term mortality, even in uncomplicated cases. Surgical interventions such as thoracic endovascular aortic repair (TEVAR) are deployed in the presence of complications, including ischemic conditions, to encourage total FL thrombosis and promote favourable aortic remodelling (i.e., FL regression).^32^

A growing body of evidence implicates haemodynamic quantities such as wall shear stress (WSS),^36^ helical and rotational flow,^15^ and FL pressure^40^ in the onset and progression of AD. Haemodynamic analysis may therefore offer predictive capabilities to support clinical decision-making. Computational fluid dynamics (CFD) and 4D-flow MRI (4DMR) are two prevalent analysis modalities. While the accuracy of CFD simulations relies heavily on modelling assumptions such as the patient-specificity of boundary conditions,^2^ 4DMR is limited in spatio-temporal resolution (≈ 1.5–3 mm in the aorta^12^) and is subject to imaging errors that result in substantial uncertainties in flow rate measurement,^24^ WSS^39^ and velocity.^12^ Informing and validating CFD with 4DMR data offers a favourable compromise between accuracy and resolution, especially where small-scale anatomical features like dissection tears are of interest.

The number of vessels branching from the FL has been significantly associated with failed FL regression^25^ and aneurysmal development^26^ in AD. Although patient-specific simulations with all major branches are routine, studies including minor aortic branches such as the segmental arteries remain scarce. The segmental branches include the intercostal, subcostal and lumbar arteries, which branch from the dorsal descending thoracic and abdominal aorta in pairs. These small (1–4.2 mm diameter^16,23^) but numerous vessels form part of an extensive collateral network of vessels supplying the spinal cord and other vital tissues and have been reported to accept between 7 and 21% of cardiac output (CO) in healthy subjects.^23,30^

The segmental branches play a complex role in TBAD. Independently of other FL branching vessels, their patency has been associated with negative outcomes including aneurysmal development.^29^ Conversely, segmental patency has been indicated as a significant protective factor in TBAD.^29^ To complicate matters, occlusion or removal of the segmental arteries during endovascular or surgical treatments for TBAD is currently attributed as the cause of postoperative paraplegia due to spinal cord ischemia (SCI).^37^

To date, minor branches have virtually always been neglected in simulations of the aorta due to limitations in medical image resolution, computational power, and a lack of available research on appropriate boundary conditions. Segmental arteries have been included as structural supports in aortic fluid–structure interaction (FSI) studies^13,19^ but without haemodynamic assessment. The inferior mesenteric artery (IMA) and a selection of FL-branching intercostal arteries were recently included in a TBAD simulation.^3^ However, TL branches were excluded and boundary conditions were not patient-specific, which has introduced large simulation errors in previous studies of TBAD.^2^ To the authors’ knowledge, no patient-specific CFD simulations of human aortae with segmental arteries have been reported to date.

The inclusion of minor branches and their flow loss will likely affect WSS and pressure distributions in simulations of TBAD. The magnitude of this effect may have implications for using haemodynamic markers to aid clinical decisions. In this study, we report a technique that simulates all pairs of segmental arteries in a patient-specific TBAD case. We evaluate their haemodynamic impact by comparing simulations with and without them, using 4DMR data and brachial pressure measurements to inform a patient-specific three-dimensional, three-component inlet velocity profile and three-element Windkessel outlet boundary conditions.

## Materials and Methods

### Clinical Data

**Figure 1:**
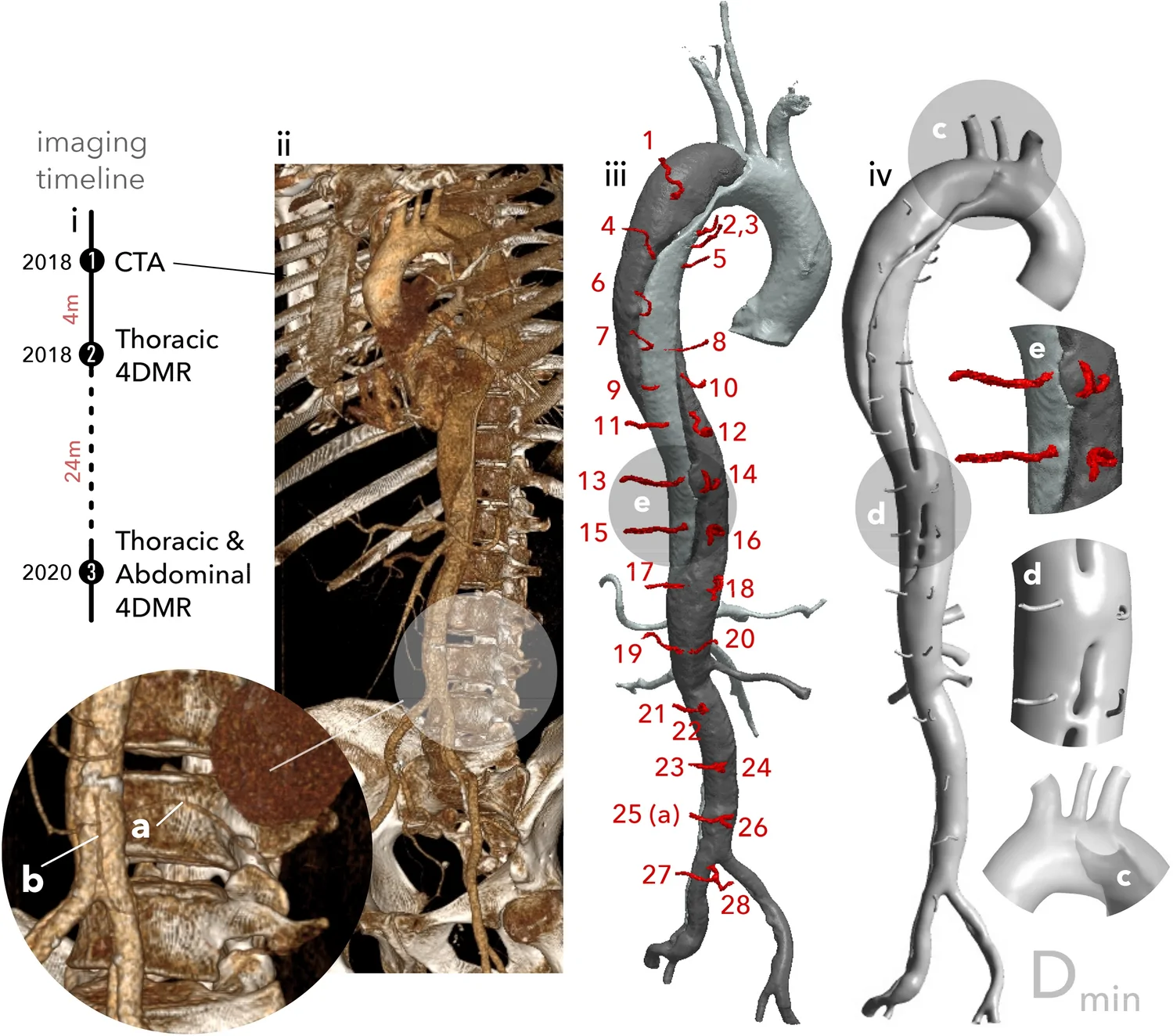
A schematic of the medical imaging timeline is shown top left (i). A 3D rendering of the CTA data is shown to its right (ii) with a detail view indicating a lumbar segmental branch (**a**) and the inferior mesenteric artery (IMA) (**b**). The baseline segmentation is shown with minor branches numbered (except the IMA, which is on the reverse side) before their reconstruction (iii). The vessel marked (**a**) in the CTA rendering detail is indicated as branch 25. A detail view (**e**) provides a closer view of the first re-entry tear and minor branches before reconstruction and smoothing. The final $D_{\min }$ domain is shown to the right (iv), with detail views (**c**) and (**d**) showing the primary entry tear (**c**) and first re-entry tear and reconstructed branches (**d**).

Computed tomography angiography (CTA) data from a 56-year-old male patient with chronic TBAD were acquired using a Siemens SOMATOM Definition Flash (Siemens Healthcare GmbH, Erlangen, Germany) with an isotropic spatial resolution of 0.5 mm. A 3D rendering of this CTA data is shown in Fig. 1ii. Four months later, 4DMR data of the thoracic aorta were acquired using a Siemens Aera 1.5T with a spatial resolution of 2.25 × 2.25 × 3.00 mm, a velocity encoding (VENC) of 150 cm/s and 16 timeframes across the cardiac cycle. Two years after the initial 4DMR data acquisition, a second set of thoracic 4DMR data and a first set of abdominal 4DMR were acquired using the same imaging settings. Thoracic and abdominal aortic 4DMR data are acquired separately as their combined volume is too large to be captured in a single scan. The medical imaging timeline is shown schematically in Fig. 1i. All medical data were acquired at Inselspital, Bern, Switzerland under ethical approval from the Local Institutional Review Board (ID 2019-00556). Area measurements from each thoracic 4DMR dataset were extracted at 5 mm increments along the FL to measure FL dilatation over the 2-year period. Throughout the thoracic aorta, a mean dilatation of 35.3% was observed, with a maximum area increase of 72.5% occurring in the proximal thoracic aorta near the first re-entry tear. A heart rate of 94 beats per minute was extracted from the 4DMR data and a single brachial measurement of 138/81 mmHg was available, obtained near the first 4DMR acquisition.

The dissection begins at the bifurcation of the left subclavian artery with a large PET ≈18 mm in diameter, shown in Fig. 1c. The dissection extends helically to the right external iliac and left iliac artery. The FL is fully patent with twenty identifiable communications with the TL, the first and largest of which is located at the bifurcation of the T9 intercostal branches, as shown in Fig. 1d. Fourteen pairs of segmental arteries and the IMA are patent and visible on CTA data, as shown numbered in Fig. 1iii.

### Segmentation and Registration

**Figure 2:**
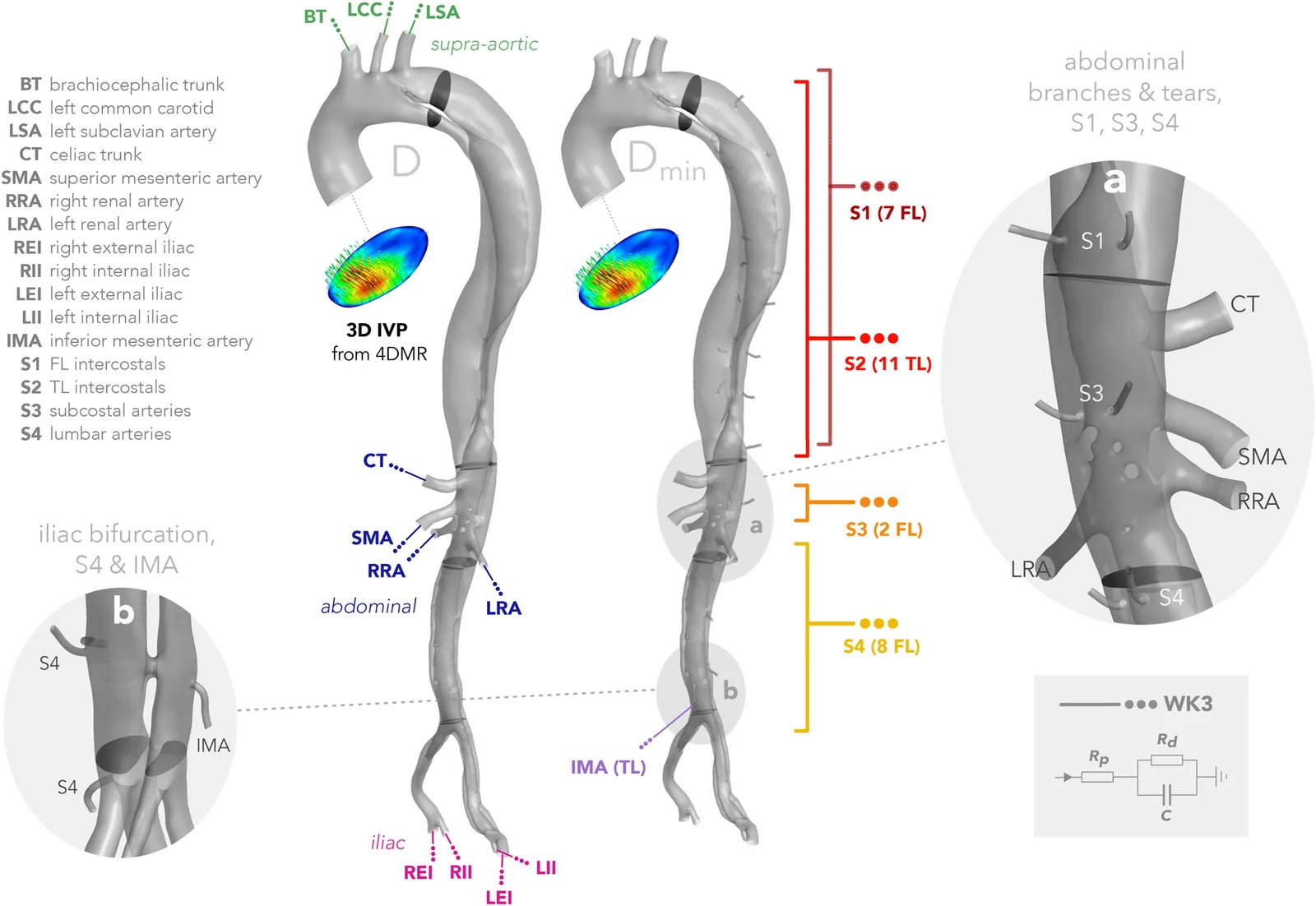
A schematic diagram of the inlet and outlet boundaries for case *D* (excluding segmental branches) and case $D_{\min }$ (including segmental branches). Each group of major branches are indicated on *D*, while each group of minor branches are indicated on $D_{\min }$. The branch abbreviations are indicated top left, while the symbolic representation of the three-element Windkessel is depicted at the bottom right. Detail view (**a**) shows the major abdominal branches, two S1 branches, both S3 and two S4 minor branches, and the variety of luminal tears located at the level of the renal arteries. Detail view (**b**) shows four S4 branches and the inferior mesenteric artery (IMA) near the iliac bifurcation. Case-specific values of flow rate, simulated flow rate, and WK3 parameters at each outlet are shown in Table 1.

The computational domain was segmented from CTA data and non-rigidly registered onto the thoracic 4DMR domain from the data acquired four months later. CTA data was used for the initial segmentation due to its superior resolution, while the registration was performed so that the overall aortic shape matched that observed in the 4DMR data during validation; the position of the patient in the scanner can greatly change the shape and hence the flow distribution within the aorta. Any FL dilatation during the 4 months between CT and 4DMR scans was reconciled during non-rigid registration. The full extent of the dissection and all major branches were included. Two otherwise identical domains were then produced: one with minor branches, $D_{\min }$, and one without minor branches, *D*, as shown in Fig. 2. The segmentation and registration process is described in detail in Supplementary Material SM1.

### Meshing

Unstructured tetrahedral volume meshes were generated using Fluent Mesh (ANSYS Inc., PA, USA). A mesh independence study was carried out using grid convergence index (GCI)^10^ to determine the appropriate mesh sizing, as described in Supplementary Material SM2. The final meshes for *D* and $D_{\min }$ contained 3.48 and 4.06 M elements, using ten near-wall layers and a first cell height corresponding to mean and peak y+ of 0.83 and 3.73 at peak systole.

### Patient-Specific Inlet Velocity Profile

Three-component velocity data was extracted from the thoracic 4DMR data on the same plane as the CFD inlet to apply a patient-specific three-dimensional, three-component inlet velocity profile. As the inlet plane moves in space and time due to aortic compliance and heart movement, an algorithm was developed in MATLAB (MathWorks, Natick, MA, USA) to dynamically map the 4DMR inlet onto the static CFD inlet, and to interpolate the 4DMR data in time to match the CFD timestep. Spatial interpolation of the inlet data onto the CFD mesh was performed by the CFD solver, ANSYS CFX 2020 R2 (ANSYS Inc., PA, USA).

### Outlet Boundary Conditions

#### Overview

Three-element Windkessel (WK3) outlet boundary conditions were applied to reconstruct physiological and patient-specific flow and pressure conditions in the aorta. Rather than using individual WK3s at every minor branch outlet, they were grouped based on location and connected jointly to a single WK3, as shown schematically in Fig. 2. This grouping served to make the tuning and simulation setup less cumbersome and may facilitate a more physiological distribution of flow amongst the branches; the minor branches are all connected to an extensive collateral network *in vivo*, so without knowledge of patient-specific individual minor branch flow rates, applying a common pressure outlet condition in groups may make more physiological sense than constraining the individual flows.

The 7 intercostals in S1 all branch from the FL while the 11 S2 intercostals all branch from the TL. S3 consists of a single pair of subcostal arteries bifurcating from the FL. S4 comprises four pairs of lumbar arteries that also bifurcate from the FL and the IMA, originating from the TL, was provided with its own WK3 outlet.

#### Major Branches

A target mean flow rate ${\overline{Q}}_{\text {B}}$ is needed at any major branch *B* to tune patient-specific WK3 parameters. Mean measured flow rates, ${\overline{Q}}'_{\text {B}}$, were extracted from 4DMR at each major branch using GTFlow (Gyrotools LLC, Zurich, CH). Within a given group of major branches, Γ, these measurements were used to establish the proportion of flow, $\phi _{\text {B}}$, leaving the branch:

1 $$\phi _{\text {B}} = \frac{{\overline{Q}}_{\text {B}}'}{\sum _{\varGamma }{\overline{Q}'}}$$

where the denominator is the sum of the measured branch flow rates in Γ, for example at the supra-aortic branches, ${\overline{Q}}'_{\text {BT}} + {\overline{Q}}'_{\text {LCC}} + {\overline{Q}}'_{\text {LSA}}$. To reduce the impact of measurement uncertainties due to image resolution, the target mean flow rate at each branch was then calculated as:

2 $${\overline{Q}}_{\text {B}} = \phi _{\text {B}} \cdot {\overline{Q}}'_{\varGamma }$$

where ${\overline{Q}}_{\varGamma }'$ is the mean flow loss measured between planes intersecting the aorta upstream and downstream of group Γ. The supra-aortic and abdominal flow rates were extracted from thoracic 4DMR while iliac flows were extracted from the abdominal 4DMR data, assuming that SV did not change substantially in the two years between acquisitions.

#### Minor Branches

Since the minor branches are smaller than the resolution of 4DMR, and their flow loss is of a similar order to 4DMR uncertainty, we used Doppler ultrasound measurements of the individual segmentals and the IMA from healthy subjects^18,23^ to derive ${\overline{Q}}_{\text {b}}$ at a given group of minor branches, *b*. See Supplementary Material SM4 for further details. All target outlet flow rates, ${\overline{Q}}$, and their corresponding WK3 parameters $R_{{\text {WK}}3}^{\text {tot}}$, ρ, and $C_{{\text {WK}}3}$ are shown in Table 1, where $R^{\text {p}}_{{\text {WK}}3} = \rho R_{{\text {WK}}3}^{\text {tot}}$ is the proximal WK3 resistance, and $R^{\text {d}}_{{\text {WK}}3} = (1-\rho ) R_{{\text {WK}}3}^{\text {tot}}$ the distal resistance. The final simulated flow rates, ${\overline{Q}}_{\text {CFD}}$ are also shown.

**Table 1:** Target (${\overline{Q}}$) and simulated (${\overline{Q}}_{\text {CFD}}$) mean outlet flow rates and WK3 parameters for each domain *D* and $D_{\min }$.

*D*	${\overline{Q}}$	${\overline{Q}}_{\text {CFD}}$	$R^{\text {tot}}_{{\text {WK}}3}$	ρ	$C_{{\text {WK}}3}$	$D_{\min }$	${\overline{Q}}$	${\overline{Q}}_{\text {CFD}}$	$R^{\text {tot}}_{{\text {WK}}3}$	ρ	$C_{{\text {WK}}3}$
	mL/s	mL/s	mmHg/mL/s		mL/mmHg		mL/s	mL/s	mmHg/mL/s		mL/mmHg
BT	17.10	*17.69*	5.77	0.030	0.235	${\text {BT}}_{\min }$	17.10	*17.39*	5.77	0.030	0.235
LCC	3.84	*3.97*	25.67	0.030	0.053	${\text {LCC}}_{\min }$	3.84	*3.88*	25.67	0.030	0.053
LSA	6.69	*6.98*	14.75	0.030	0.092	${\text {LSA}}_{\min }$	6.69	*6.80*	14.75	0.030	0.092
						S1 (FL, 7)	$5.50^{\dagger}$	*5.09*	17.93	0.056	0.075
						S2 (TL, 11)	$7.97^{\dagger}$	*7.43*	12.37	0.056	0.109
						S3 (FL, 2)	$2.11^{\dagger}$	*1.89*	46.79	0.056	0.029
CT	16.31	*16.14*	6.05	0.056	0.223	${\text {CT}}_{\min }$	12.38	*12.39*	7.97	0.056	0.170
SMA	14.09	*13.86*	7.00	0.056	0.192	${\text {SMA}}_{\min }$	10.70	*10.72*	9.22	0.056	0.147
LRA	16.59	*15.63*	5.94	0.280	0.227	${\text {LRA}}_{\min }$	12.20	*12.10*	8.09	0.280	0.167
RRA	12.64	*12.25*	7.80	0.280	0.173	${\text {RRA}}_{\min }$	9.20	*9.23*	10.72	0.280	0.126
						S4 (FL, 8)	$4.29^{\dagger}$	*3.98*	23.01	0.056	0.059
						IMA (TL)	$1.93^{\ddagger}$	*1.71*	51.12	0.056	0.026
LEI	9.61	*9.60*	10.26	0.056	0.131	${\text {LEI}}_{\min }$	6.77	*6.87*	13.57	0.056	0.093
LII	4.76	*4.76*	20.72	0.056	0.065	${\text {LII}}_{\min }$	3.35	*3.38*	27.39	0.056	0.046
REI	5.21	*5.41*	18.95	0.056	0.071	${\text {REI}}_{\min }$	3.40	*3.46*	26.97	0.056	0.047
RII	3.01	*3.17*	32.79	0.056	0.041	${\text {RII}}_{\min }$	1.97	*1.99*	46.67	0.056	0.027

Outlet abbreviations are summarised in Fig. 2. Segmental flow rates indicated with a^†^ are derived from Koyanagi *et al*.^23^ and the IMA flow rate with a^‡^ is extracted from Erden *et al*.^18^ See Supplementary Material SM4 for further details. ρ is the proportion of $R_{\text {tot}}$ assigned to the proximal resistor in each WK3, with the remaining amount assigned to the distal resistor. The number of individual branches in each grouped WK3 is indicated in parentheses for the relevant outlets, and their locations are shown schematically in Fig. 2

#### Pressure Targets

Finally, target systolic and diastolic pressures, $P_{\text {s}}$ and $P_{\text {d}}$ are required to tune patient-specific WK3 conditions. We derive these using the patient brachial measurements $P_{\text {s}}^{\prime }$ and $P_{\text {d}}^{\prime }$, where ^′^ indicates the measured value. Diastolic pressure remains relatively constant throughout the arterial tree, so $P_{\text {d}} \approx P_{\text {d}}^{\prime }$. However, systolic pressure tends to increase peripherally, with $P_{\text {s}} \approx 0.83P_{\text {s}}^{\prime } + 0.15P_{\text {d}}^{\prime }$.^35^. Pressure targets and measurements, and their simulated values, are shown in Table 2.

**Table 2:** Measured, target and simulated systolic and diastolic pressures and pulse pressure ($P_{\text {p}}$).

	Pressure (mmHg)		
	Systolic	Diastolic	Pulse
Brachial measurement ($P^{\prime }$)	138	81	57
Aortic inlet target (*P*)	$127^{\dagger }$	81	46
D inlet	127	80	47
$D_{\min }$ inlet	128	83	44

^†^Target systolic pressure is calculated using an empirical formula^35^

With target inlet pressure and mean outlet flow rates determined, WK3 parameters were tuned *via* a lumped parameter model of the aorta using our previously developed technique.^6,31^

### Simulation

The three-dimensional incompressible Navier–Stokes and continuity equations were solved numerically using the finite-volume solver ANSYS CFX 2020 R2. Walls were assumed to be rigid, both for computational efficiency and due to a lack of Cine-MRI data to tune patient-specific aortic compliance with our existing techniques.^6,31^

Blood was modelled as a non-Newtonian fluid using the Carreau–Yasuda viscosity model with empirical constants from Tomaiuolo *et al*.^33^ and a density of 1056 kg/m^3^. Using the Reynolds number (*Re*) for pulsatile cardiovascular flow,^28^ the nominal shear rate,^9^ and the peak inlet velocity from 4DMR, a peak $Re_{\text {p}}$ of 11646 was estimated, which substantially exceeds the critical^28^ $Re_{\text {c}}$ of 6959. When calculated using the diameter of the PET as a characteristic length scale, along with its associated peak velocity, the $Re_{\text {p}}$ still exceeded $Re_{\text {c}}$. As such, the k–ω shear stress transport (SST) Reynolds-averaged Navier-Stokes (RANS) turbulence model was employed due to its ability to predict the onset and amount of flow separation under adverse pressure gradients, which are observed in the region of jet flow through the PET, for example. Low turbulence intensity (1%) was applied at the inlet and outlets.^22^ The use of RANS models is open to question in the context of pulsatile blood flow, even with transition to turbulence. An equivalent set of laminar flow simulations were carried out to ensure that the study conclusions were upheld. This analysis can be found in Supplementary Material SM3.

Transient simulations with timesteps of 1 ms were solved using the implicit, second-order backward-Euler method using a root-mean-square residual value of $10^{-5}$ for all equations within each timestep. Simulations were run until <1% change was observed in systolic and diastolic pressures between cycles. After initialisation with an approximate flow field, 6 cycles were required in each simulation to reach cyclic periodicity.

### Haemodynamic Analysis

Flow rate, pressure, velocity magnitude and WSS indices were analysed in each simulation to compare haemodynamics in cases *D* and $D_{\min }$ and investigate the impact of minor branch flow loss. CFD data was compared with 4DMR velocity and flow rate measurements where possible.

The net instantaneous flow rate, *Q*, was extracted from CFD and 4DMR on selected analysis planes throughout the dissection. On a given plane Φ, perpendicular to and enclosing both TL and FL, *Q* was defined at each timestep as:

3 $$\begin{aligned}&\overrightarrow{{\mathbf {v}}} = {\mathbf {v}}\cdot \overrightarrow{{\mathbf {n}}}, \end{aligned}$$

4 $$\begin{aligned}&Q(t) = \int _{\varPhi }=\overrightarrow{{\mathbf {v}}} {\text {d}}A, \end{aligned}$$

where ${\mathbf {v}}={\mathbf {v}}(x,y,z,t)$ is the local velocity magnitude, $\overrightarrow{{\mathbf {n}}}$ is the plane normal, and *A* is the cell/voxel area. Next, *Q* was decomposed into forward and reverse flow rates to assess the degree of retrograde flow in the FL, which may be linked with TBAD progression.^14^ At any given timestep, forward and reverse flow regions $\varPi _{\text {F}}$ and $\varPi _{\text {R}}$ were defined on a given FL plane, Π, as follows:

5 $$\begin{aligned}&\varPi _{\text {F}} (t) = \{{\mathbf {x}}(x,y,z) \in \varPi : \overrightarrow{{\mathbf {v}}} > 0 \}, \end{aligned}$$

6 $$\begin{aligned}&\varPi _{\text {R}} (t) = \{{\mathbf {x}}(x,y,z) \in \varPi : \overrightarrow{{\mathbf {v}}}< 0\}, \end{aligned}$$

where ${\mathbf {x}}$ is the location of each intersected mesh element on plane Π. In this way, the forward and reverse flow rates, $Q_{\text {F}}$ and $Q_{\text {R}}$, could be computed at each time point using Eq. (4) over $\varPi _{\text {F}}$ and $\varPi _{\text {R}}$, respectively. Instantaneous and cycle-averaged reverse/forward flow ratios, *R*/*F* and $\overline{R/F}$, were then calculated as follows:

7 $$\begin{aligned}&R/F = \frac{Q_{\text {R}}}{Q_{\text {F}}}, \end{aligned}$$

8 $$\begin{aligned}&\overline{R/F} = \frac{1}{T}\int _{t}^{t+T}\frac{Q_{\text {R}}}{Q_{\text {F}}} {\text {d}}t, \end{aligned}$$

where *t* is the start time of the final cycle and *T* is the cycle period. FL ejection fraction (FLEF), a predictor of aortic growth rate,^8^ was also assessed by evaluating $\overline{R/F}$ across the PET.

To compare pressure distributions between *D* and $D_{\min }$, we analysed transmural pressure (TMP) throughout the dissection. ${\text {TMP}}_{\text {mean}}$, sometimes referred to as luminal pressure difference (LPD),^38^ provides a quantification of luminal interaction and is defined as:

9 $${\text {TMP}}_{\text {mean}} = \frac{1}{T}\int _{t}^{t+T}P_{\text {TL}}(t) - P_{\text {FL}}(t) {\text {d}}t,$$

where $P_{\text {TL}}$ and $P_{\text {FL}}$ are measurements of area-averaged static pressure across the indicated plane acquired at each timestep. The peak instantaneous magnitudes of TMP across the cardiac cycle were also extracted. We will use ${\text {TMP}}_{\max }$ to refer to the peak magnitude of TMP at each location, which may be positive or negative.

To assess the impact of minor branches on WSS distributions, time averaged WSS (TAWSS), oscillatory shear index (OSI), and endothelial cell activation potential (ECAP) were computed using every fifth timestep (every 5 ms) of the final cycle as follows:

10 $${\text {TAWSS}} = \frac{1}{T}\int _t^{t+T} |\boldsymbol{\tau }|{\text {d}}t,$$

11 $${\text {OSI}} = \frac{1}{2}\left( 1-\frac{\left| \frac{1}{T}\int _t^{t+T} \boldsymbol{\tau }{\text {d}}t\right| }{\frac{1}{T}\int _t^{t+T} |\boldsymbol{\tau }|{\text {d}}t}\right) ,$$

12 $${\mathrm{ECAP}} = \frac{{{\mathrm{OSI}}}}{{{\mathrm{TAWSS}}}},$$

where $\boldsymbol{\tau }$ is the instantaneous WSS vector.^21^

## Results

Simulated $P_{\text {d}}$ and $P_{\text {s}}$ and mean flow rates across the cardiac cycle at each outlet are shown against their target values in Tables 1 and 2. In both cases, pressures and mean flows were predicted within 3% of target values. Supra-aortic branch flows differed < 2.5% between *D* and $D_{\min }$, indicating that comparable flow conditions were successfully applied in each simulation. Results were analysed on 12 planes, a–l, spaced evenly along the dissection as shown in Fig. 4. 4DMR data at plane l is unreliable as each lumen is only one voxel across, thus simulations will be compared here, but not against 4DMR data.

### Flow Rate

**Figure 3:**
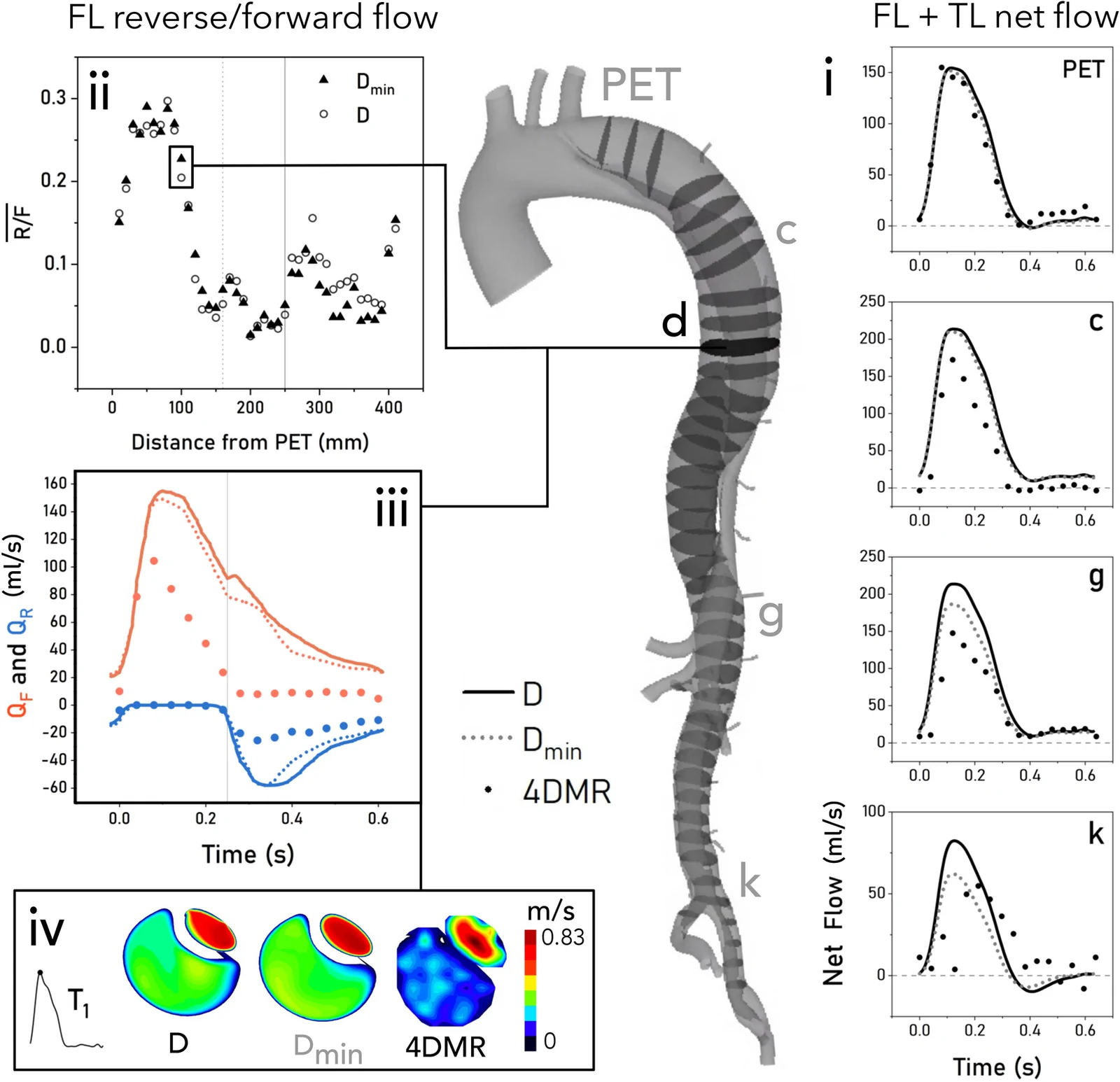
Flow rate analysis of *D*, $D_{\min }$ and 4DMR throughout the dissection with a diagram of flow analysis planes at 10 mm increments. Plots on the right hand side indicate the total plane flow (TL + FL) through the primary entry tear (PET) and selected planes in CFD and 4DMR. Plots on the left side provide forward and reverse flow analysis in the FL only. The top left plot shows reverse/forward flow ratio along the dissection, extracted from all planes shown on the diagram. The bottom left two plots show forward and reverse flow across the cycle from CFD and 4DMR at plane *D*, indicated, along with velocity contours from this plane at peak systole indicating poor FL velocity signal in 4DMR. Solid/dashed lines correspond to $D/D_{\min }$ respectively while 4DMR is represented as points.

Minor branch outflow caused a progressive reduction in the total flow rate along the aorta, leading $D_{\min }$ to agree favourably with 4DMR, as shown in Fig. 3i. 18% of the total SV was lost through these minor branches, in line with measured values.^23^ It is important to note that 4DMR suffers from numerous sources of uncertainty. Poor accuracy is observed in low-velocity regions due to a low signal-to-noise ratio.^24^ Depending on spatial resolution and the choice of VENC, 4DMR is also prone to underestimating peak velocity^34^ and overestimating luminal diameter.^12^ As a result, uncertainties in velocity and flow rate of 20–110% have been reported in single-VENC 4DMR in healthy ascending aortae.^12,24^ In this study, measurement uncertainty in 4DMR data was not readily quantifiable. Additionally, the magnitude and source of measurement errors differ in each lumen due to their relatively different sizes and velocity ranges. For these reasons, we use the total plane flow rate in Fig. 3i to illustrate the effect of minor branches, rather than to quantify the accuracy of the simulations.

Including minor branches reduced net flow through the PET by 7.9%, drawing more flow into the TL. A progressive delay in peak flow rate is observed in the 4DMR data in Fig. 3i, but not in CFD, due to the rigid-wall assumption.

Examining forward and reverse flow dynamics in the FL, the evolution of $\overline{R/F}$ along the dissection is similar in both simulations, as shown in Fig. 3ii. Flow reversal reaches a maximum in the proximal FL, dropping rapidly to the first re-entry tear (x=160 mm, dotted line) and reaching its minimum near the abdominal aortic branches (x=250 mm, solid line). The inclusion of minor branches increased $\overline{R/F}$ by only 0.7% up to the re-entry tear and 0.1% between the re-entry tear and the abdominal branches. $\overline{R/F}$ increases again in the abdominal aorta in both cases, where minor branches reduced $\overline{R/F}$ by 2% on average. Measured from 4DMR, FLEF was only 2.1% in this patient and simulated as ≈ 0.1% in each case.

Observing the temporal dynamics of forward and reverse flow on plane d in Fig. 3iii, reverse flow increases after valve closure (t=0.25 s). While the inclusion of minor branches does not greatly affect reverse flow rates, it does cause an earlier and more rapid decay in forward and reverse flow. Comparing simulated forward and reverse flow at plane d with 4DMR, similar flow waveforms are observed but with lower magnitudes in 4DMR. However, velocities in the FL are underestimated as low-velocity regions suffer from poor imaging signal, an effect which can be seen in Fig. 3iv.

### Transmural Pressure

**Figure 4:**
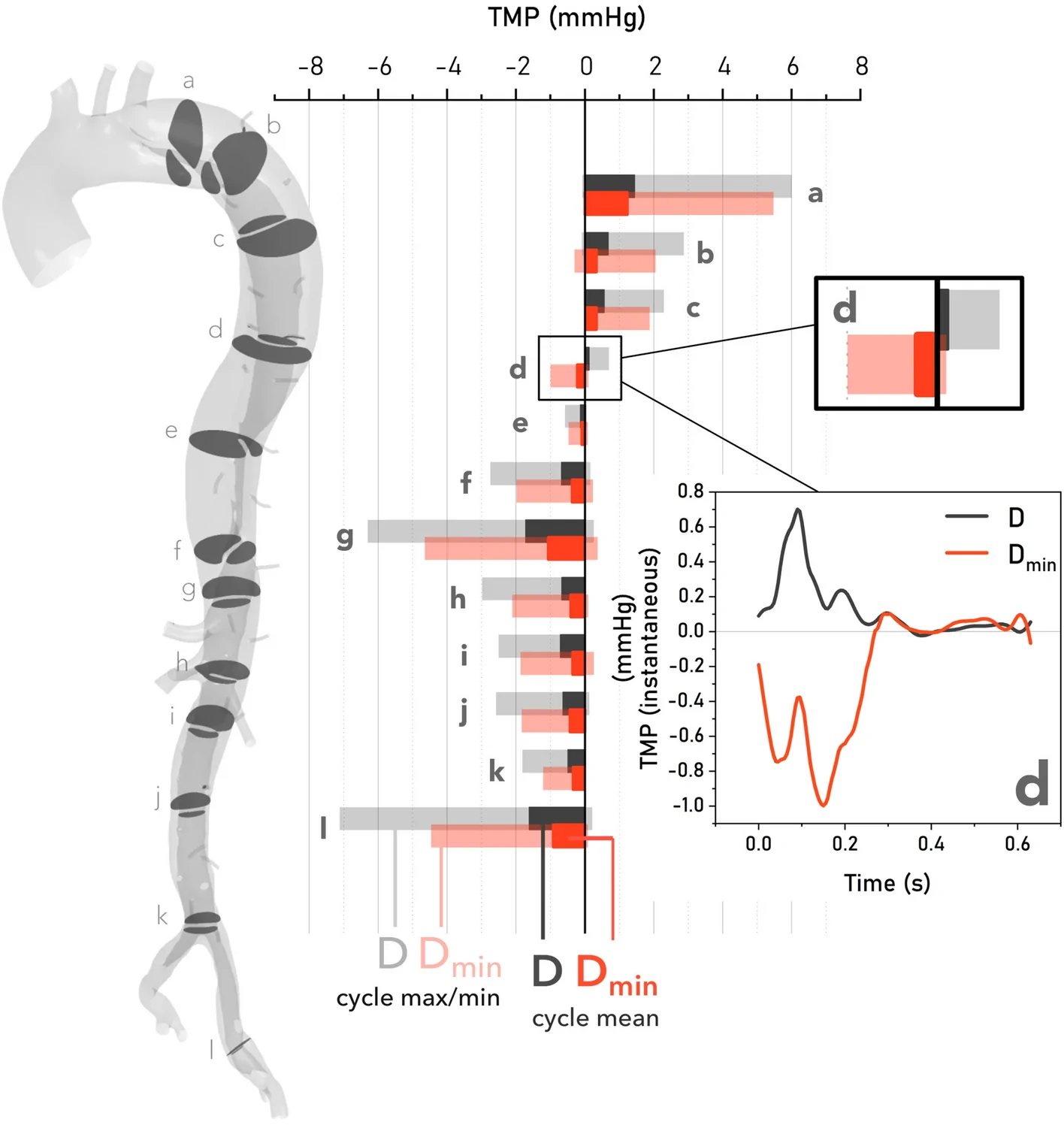
Vertical histogram comparing maximum and cycle-averaged (mean) transmural pressure (TMP) in cases *D* and $D_{\min }$ along the full length of the dissection. Maximum values are indicated by the lighter, transparent bars, while mean values are indicated by the darker, solid bars. $D_{\min }$ provides lower magnitudes of mean and maximum TMP everywhere except plane *D*, which lies proximally to the first re-entry tear at plane e, as shown in the inset detail.

Figure 4 shows the evolution of ${\text {TMP}}_{\text {mean}}$ on indicated planes along the dissection. The maximum and minimum instantaneous values of TMP across the cycle are shown as transparent bars to indicate the range of TMP experienced at each location. In both simulations, the TL is more pressurised in the proximal thoracic aorta, planes a–c, while the FL is more pressurised everywhere beyond the first re-entry tear, planes e–l. ${\text {TMP}}_{\text {mean}}$ and ${\text {TMP}}_{\max }$ are reduced by an average of 61% in $D_{\min }$ and are larger in magnitude everywhere except plane d.

Plane d on Fig. 4, located ≈ 35 mm proximal to the first re-entry tear, is the only plane where the sign of TMP_mean_ differs between cases, being negative in $D_{\min }$ and positive in *D*. This indicates that the first balance point (FBP), defined as the first location where ${\text {TMP}}_{\text {mean}} = 0$, occurs more proximally in $D_{\min }$. Plane *d* is also the only location where TMP_max_ is higher in $D_{\min }$. Furthermore, this plane lies in the region of maximal FL growth. The temporal variation of TMP at plane d is also shown inset in each case, illustrating that pressure dynamics between *D* and $D_{\min }$ differ substantially at this location.

### Velocity

**Figure 5:**
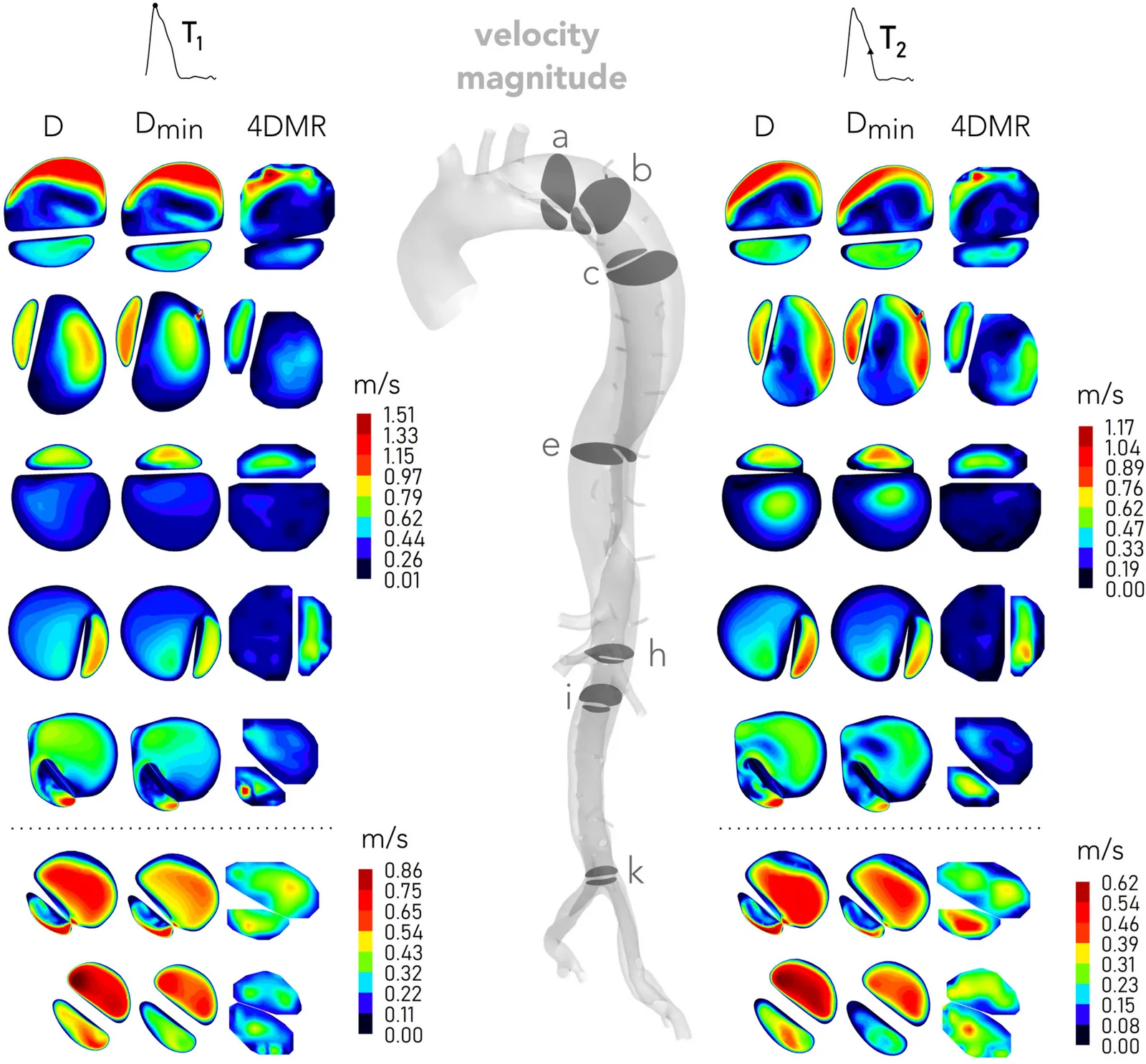
Simulated and measured velocity magnitude contours throughout the dissection at peak systole ($T_{1}$) and late systole ($T_{2}$). Contours from *D* and $D_{\min }$ (left, centre columns) are compared with contours of measured velocity magnitude from 4DMR (right column), as indicated. Contour scales are matched between planes a–h and are adjusted on planes i–k, separated with a dotted line. Note that planes are not exactly to scale. Contours from omitted planes can be found in Supplementary Material SM5.

Velocity magnitude contours from simulations and 4DMR are compared on selected planes at peak systole ($T_{1}$) and late systole ($T_{2}$) in Fig. 5. Contours from omitted planes can be found in Supplementary Material SM5. Although differences in velocity magnitude are observed between *D* and $D_{\min }$, as will be discussed further below, flow distributions are similar and provide excellent qualitative agreement with 4DMR throughout. High FL velocities are observed on Fig. 5 plane a as flow jets through the PET. On planes b–g, the TL reduces in size while flow beyond the PET jet disperses throughout the FL, resulting in high TL and very low FL velocities. Near plane h, three of the four major abdominal branches bifurcate from the TL, reducing TL velocity. Beyond plane i, FL velocity exceeds TL velocity as FL area reduces and flow is transferred to the TL due to negative TMP and the presence of multiple tears. These trends are reflected in both CFD and 4DMR.

Changes in velocity distribution between time instants $T_{1}$ and $T_{2}$ are most apparent on Fig. 5 planes a–c where blood jets through the PET into the FL, leading to more disorganised flow patterns as flow decelerates. On plane a, the simulated location of peak velocity shifts counterclockwise between $T_{1}$ and $T_{2}$ to more closely match 4DMR, whose distributions remain similar at both time instants. Beyond plane c, distributions do not notably change between $T_{1}$ and $T_{2}$.

Widespread low velocity regions, where 4DMR incurs high uncertainty, are observed in the FL in Fig. 5 on planes b–g. While there is excellent qualitative agreement between CFD and 4DMR, substantially higher FL velocity magnitudes are observed in CFD on these planes. Due to low spatial resolution, regions of high velocity gradients (such as the fluid boundary layer) are poorly captured by 4DMR, particularly in smaller regions. On planes h and i, for example, 4DMR resolution cannot resolve the velocity distribution observed in CFD.

**Figure 6:**
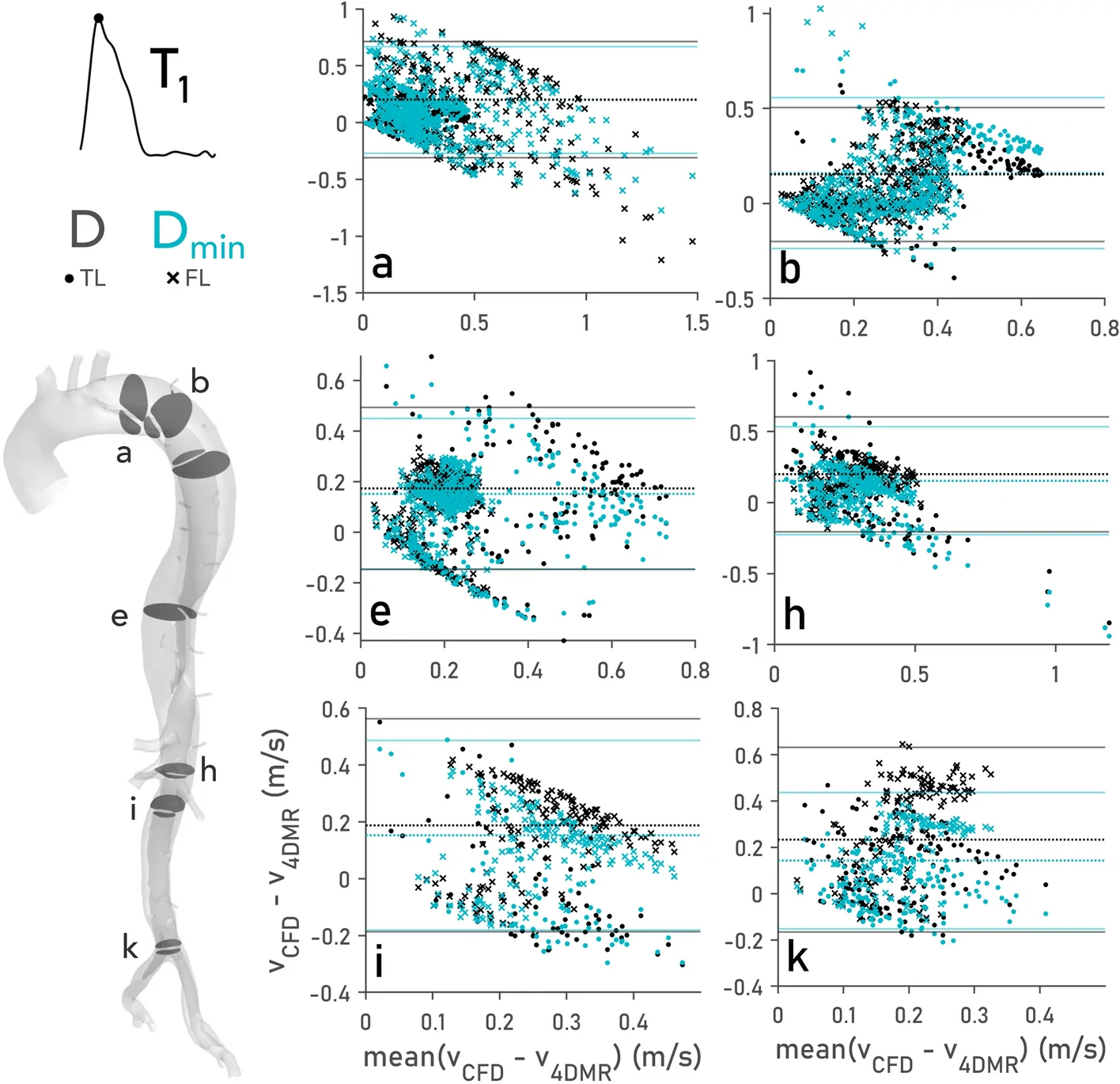
Bland–Altman plots comparing CFD and 4DMR velocity magnitude in cases *D* and $D_{\min }$ at $T_{1}$ (peak systole) on the indicated planes. Bias values [calculated as mean($v_{\text {CFD}}-v_{4{\text {DMR}}})$ in each case] are indicated by a horizontal dotted line and are assessed quantitatively in Table 3 at $T_{1}$. The limits of agreement, defined as ±1.96 standard deviations, are indicated as solid lines. Bland–Altman plots from omitted planes at $T_{1}$, and on all planes at time instant $T_{2}$, can be found in Supplementary Material SM5.

Pointwise differences in velocity magnitude between simulations and 4DMR were quantified to support qualitative flow comparisons. This data was visualised using Bland–Altman plots,^5^ as shown in Fig. 6. The mean pointwise error for each case, their bias, is shown as a dotted line while the limits of agreement, defined as ±1.96 standard deviations, are shown as solid lines. Discrepancies in peak and mean velocity magnitude between CFD and 4DMR were calculated as a percentage of mean inlet velocity at peak systole ($T_{1}$) and are provided in Table 3. Additional Bland–Altman plots for all analysis planes at $T_{1}$ and $T_{2}$ and quantitative velocity errors at $T_{2}$ can be found in Supplementary Material SM5, while details around the error calculations used in Table 3 can be found in SM6.

Bland–Altman point clouds (Fig. 6) are bounded at their lower edge where $v_{\text {CFD}}=0$, and along their upper edge by peak values of $v_{\text {CFD}}$. Relative increases and decreases in velocity magnitude between CFD cases are thus observed as an outward or inward shift from y=0 and a corresponding outward or inward shift in the limits of agreement. This effect is more clearly observed on distal planes (e.g., Fig. 6i and k) due to minor branch flow loss, where the limits of agreement differ greatly between *D* and $D_{\min }$. Where points are strongly clustered in the top left and bottom right of the plot, such as Fig. 6 planes a, h and i, qualitative differences in velocity distribution between CFD and 4DMR are more apparent. The low spatial resolution of 4DMR produces more noise in smaller vessels, resulting in a less organised point cloud structure on Fig. 6 planes i and k.

During systole, flow distributions in *D* and $D_{\min }$ are qualitatively similar, but peak and mean velocity magnitude differ substantially. Reduced flow through the PET in $D_{\min }$ yields higher TL and lower FL velocities than *D* on Fig. 5 planes a–d, resulting in higher $D_{\min }$ discrepancies on planes a–d in Table 3. Beyond plane *d*, the flow loss through the minor branches progressively reduces velocity magnitude in both TL and FL. $D_{\min }$ thus provides a closer match in peak and mean velocity on most planes beyond d, as reflected by lower average errors in Table 3; across both lumens, mean velocity was 7.9% closer to 4DMR in $D_{\min }$ than in *D*. As shown on the far-right column in Table 3 greatest improvements in velocity agreement with 4DMR were observed on planes g, 26.4%, k, 22.9%, and l, 45.9%.

Velocity in the minor branches reaches up to 2 m/s at peak systole, exceeding physiological values.^23^ As plane b intersects an intercostal vessel in the FL of $D_{\min }$, a high discrepancy with 4DMR can be seen in Table 3. Relative to *D*, peak velocity error was increased by 68.5% in $D_{\min }$ as shown in the right-hand column. This plane was therefore excluded from the peak discrepancy average in the FL, as indicated by a †.

**Table 3:** Discrepancies in peak and mean velocity between CFD and 4DMR expressed as a percentage of mean inlet velocity at time instant $T_{1}$.

	Pointwise velocity errors at $T_{1}$ (%)								
	Peak				Mean				Maximum difference (%)
	TL		FL		TL		FL		
Plane	*D*	$D_{\min }$	*D*	$D_{\min }$	*D*	$D_{\min }$	*D*	$D_{\min }$	$D-D_{\min }$
a	7.6	18.3	− 16.8	− 27.0	17.1	24.6	43.2	40.1	10.2
b	17.0	25.5	28.8^†^	97.3^†^	37.0	47.5	23.9	21.5	− 68.5
c	9.6	19.7	4.7	3.1	26.2	33.7	14.7	12.4	− 10.1
d	− 7.1	− 9.3	5.8	8.2	29.5	24.9	20.4	17.8	− 7.5
e	15.5	5.4	12.3	18.6	37.7	31.7	28.4	25.5	10.1
f	30.5	16.0	18.0	17.8	55.0	38.1	39.1	32.6	16.9
g	46.8	25.0	14.0	13.5	106.0	79.6	36.1	29.7	26.4
h	1.4	– 18.1	15.0	6.3	44.4	41.5	35.5	22.4	19.5
i	27.9	17.4	22.7	14.9	32.6	33.3	35.0	25.9	10.5
j	12.0	3.0	24.3	20.8	14.7	11.8	49.0	34.8	14.2
k	14.1	− 0.5	36.2	25.0	27.1	15.8	59.7	36.8	22.9
l	101.6	66.8	43.0	30.6	136.0	90.1	39.9	26.6	45.9
Average	23.1	14.1	16.3	12.0	46.9	39.4	35.4	27.2	

The average error is shown on the final row, while the maximum difference between *D* and $D_{\min }$ across peak and mean velocities are shown in the rightmost column. A † indicates values that were excluded from calculations of averages (bottom row) for reasons explained in Section “Velocity”. Further details on error calculations are provided in Supplementary Material SM6.

### Wall Shear Stress

**Figure 7:**
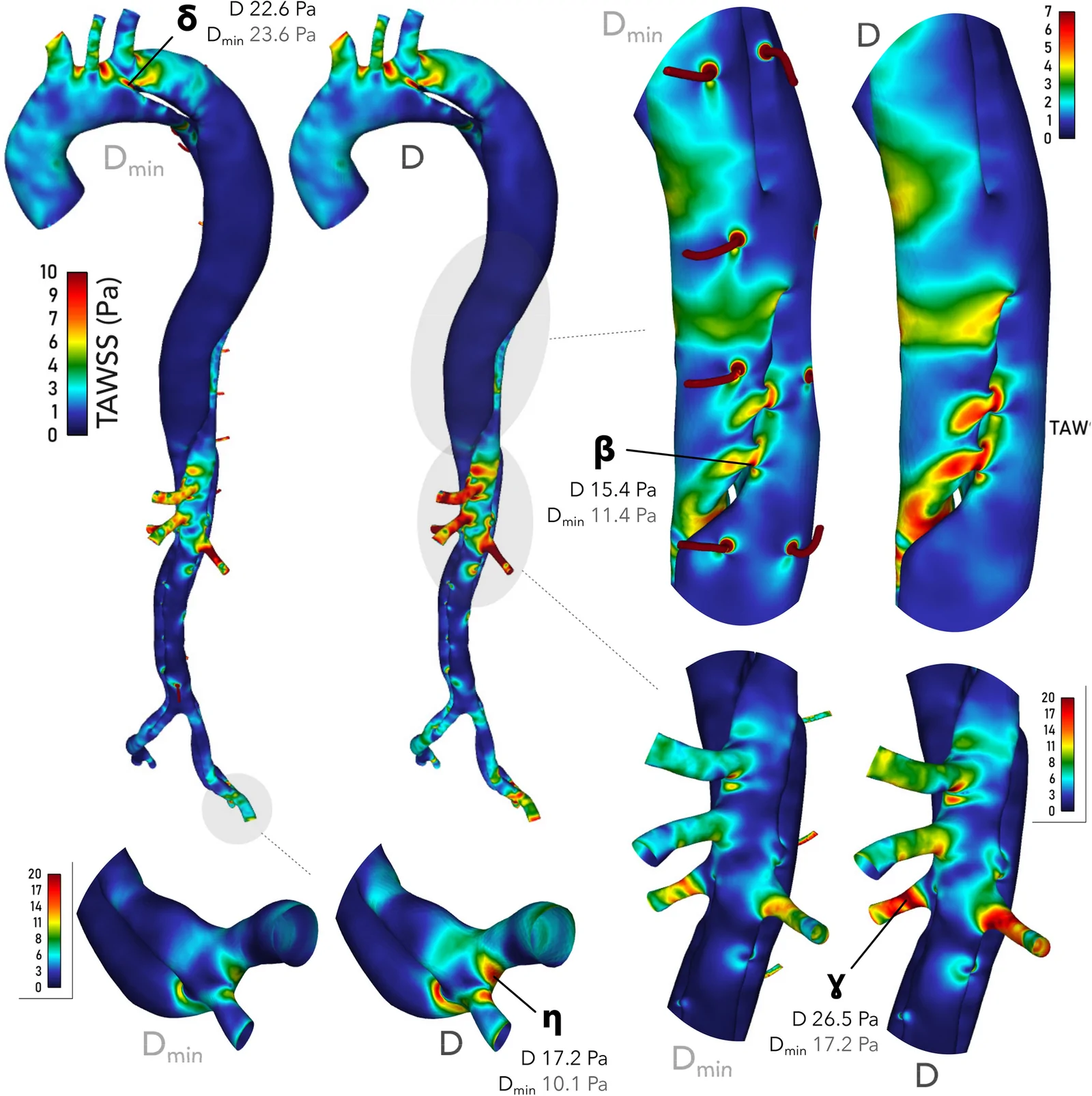
TAWSS contours in cases *D* and $D_{\min }$. δ, β, γ and η are the probe points referred to in the text. We observe similar TAWSS at the PET in both cases, but an increasing difference between them along the aorta. Point η indicates that peak TAWSS is 70% higher in *D* than $D_{\min }$ by the iliac bifurcation. Contour ranges vary between subfigures but are identical between *D* and $D_{\min }$ within each subfigure.

TAWSS in the proximal aorta and at the PET differs by less than 5% between *D* and $D_{\min }$, as shown at point δ in Fig. 7. Progressively larger differences develop between *D* and $D_{\min }$ along the dissection, at points β, γ and η, reaching a maximum of 70% at η. TAWSS is locally affected in the downstream regions surrounding each minor branch bifurcation and is high within the branches, between 20 and 40 Pa in the segmental arteries and 50–200 Pa in the IMA. These values are not physiological as the simulated velocities in these branches substantially exceed physiological values, as discussed earlier.

**Figure 8:**
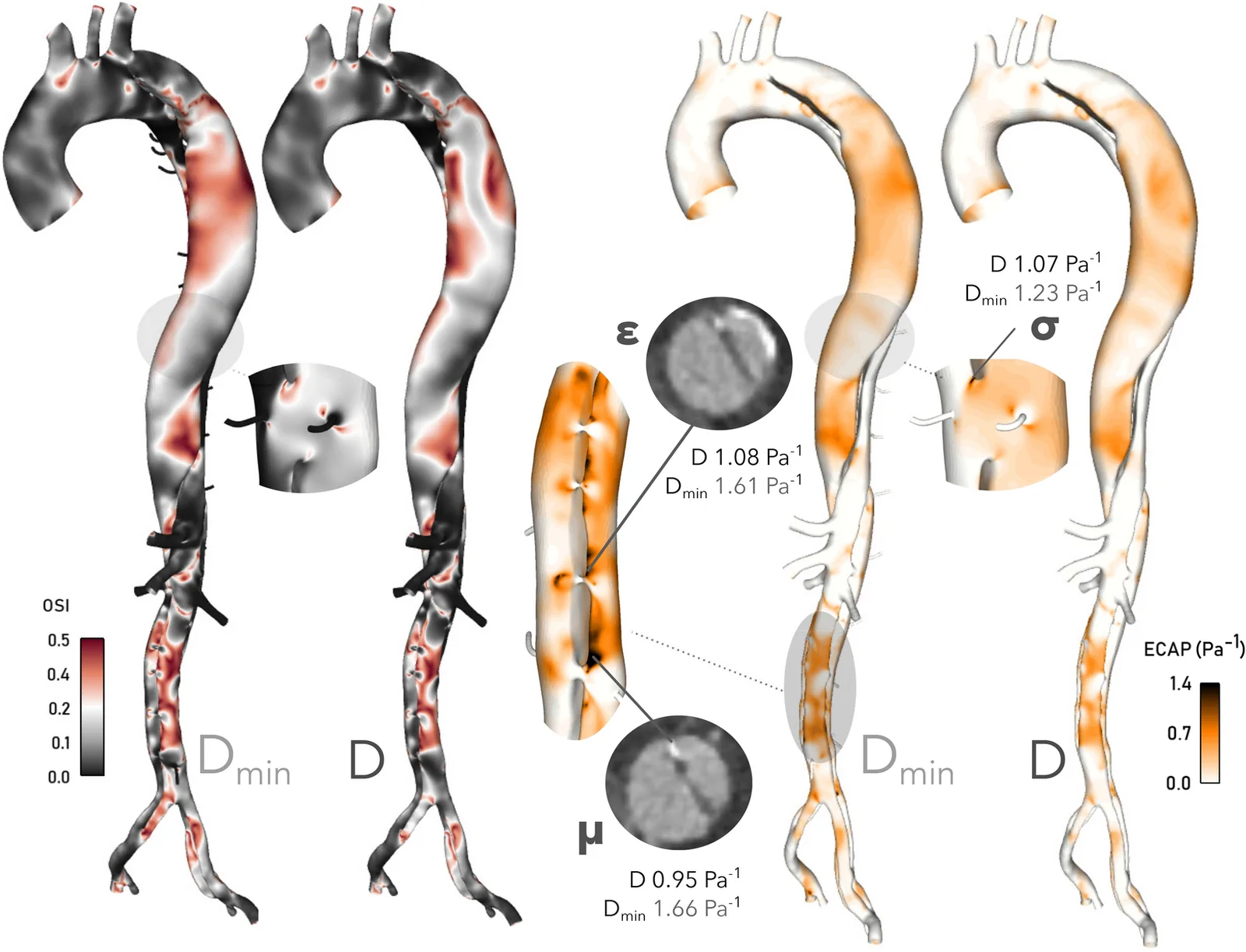
Contours of OSI and ECAP in *D* and $D_{\min }$. Probe points at ϵ, σ and μ indicate higher levels of ECAP in $D_{\min }$ throughout. CT slices at ϵ and μ indicate calcification at these sites, possibly linked to high ECAP levels. Note that ECAP contours are clipped maximally to 1.4 $\hbox {Pa}^{-1}$, the suggested threshold for intraluminal thrombus formation.^21^

As shown in Fig. 8, the inclusion of minor branches produces more widespread regions of high OSI in the thoracic FL, where FL dilatation was observed *in vivo*. On the contrary, OSI is very low in the minor branches, with localised regions of high OSI on the distal side of their bifurcations, as shown in Fig. 8. Elsewhere, OSI distributions remain qualitatively similar but with higher values of OSI observed in $D_{\min }$.

The combination of lower TAWSS and higher OSI in $D_{\min }$ lead to changes in ECAP, also shown in Fig. 8, which is a metric used to characterise the degree of thrombotic susceptibility of the vessel wall^21^ and has been linked with sites of calcification.^17^ Critical thrombotic susceptibility^21^ is defined where ECAP reaches 1.4 Pa^-1^ and above. Distributions are qualitatively similar in each case, with low values everywhere except the thoracic FL and abdominal TL. Broader regions of higher ECAP are observed in $D_{\min }$ in the thoracic FL, exceeding the threshold value only in $D_{\min }$ at point σ, shown in Fig. 8. In the abdominal TL, regions of high ECAP are more extensive in both cases. At points ϵ and μ, ECAP exceeds the threshold value, but only in $D_{\min }$. Calcification is present in the CTA data at these points, as shown in Fig. 8.

## Discussion

In this study, we developed a strategy to include minor branches in aortic CFD simulations with physiological outflows (18% of stroke volume). We have assessed their haemodynamic impact in a patient-specific simulation of uncomplicated chronic TBAD exhibiting long-term FL dilatation. Minor branch flow loss reduced velocity magnitude along the dissection, providing favourable agreement with 4DMR data. Neglecting this loss increased TAWSS by up to 70% and TMP by 61%. Minor branch flow loss also increased ECAP by up to 75%, notably in regions where aneurysmal dilatation and calcification were observed *in vivo*. These differences exceed the reported magnitude of other modelling assumptions such as wall compliance^31^ and suggest that modelling minor branch flow loss may be a vital consideration for simulation accuracy.

To our knowledge, this study represents the first analysis of the effect of minor branches on aortic haemodynamics using patient-specific boundary conditions. Only one other study,^3^ has investigated the haemodynamic impact of minor branches in TBAD, though without patient-specific boundary conditions and only including branches originating from the FL. They applied a 5% flow loss through 11 intercostal vessels and 2.2% through the IMA using fixed flow rate outlets, observing increased TAWSS around minor branch bifurcations, a 1 mmHg reduction in TMP magnitude and a reduction in TL velocity in the distal thoracic aorta, in agreement with the current study. Contrary to our findings, they observed a 2% increase in PET flow. This was attributed to the low-pressure minor branch outlets in the FL drawing additional flow through the FL. Because our study also includes TL branches, which outnumber FL branches in the thoracic aorta and carry 46% more flow, the opposite effect was observed: a 7.9% reduction in PET flow. Thus the inclusion of all minor branches, and the flow loss applied to them, appear to be an important consideration for accurate luminal flow distributions in simulations of TBAD.

With or without minor branches, ${\text {TMP}}_{\text {mean}}$ was positive (TL-dominant) in the proximal thoracic aorta and negative (FL-dominant) beyond the first re-entry tear, as reported in other cases of TBAD.^38^ However, pressure dynamics differed between cases in the proximal thoracic FL where substantial aortic growth was observed. The greater number of TL minor branches and thus their greater outflow caused FL pressure to dominate TL pressure more proximally in $D_{\min }$. This resulted in a proximal shift in first balance point (FBP), a metric which has shown potential in predicting the long-term success of TEVAR.^38^ Despite equal systolic and diastolic pressure at the inlet, neglecting minor branch flow loss increased mean and peak TMP magnitude by 61% on average with progressively greater differences along the length of the dissection.

Mean reverse/forward flow ratio, $\overline{R/F}$ was <1% higher in the thoracic aorta and 2% lower in the abdominal aorta when minor branches were included. While these changes are likely insignificant, minor branches also caused a more rapid decay in forward and reverse flow rate during diastole. Higher levels of reversed FL flow, FL ejection fraction (FLEF) and ${\text {TMP}}_{\text {mean}}$ are associated with rapid aortic growth due to their associations with elevated FL pressure.^8,42^ Despite substantial aneurysmal growth in this patient, a low-to-moderate degree of reverse flow and negligible FLEF (<2%) were observed in both simulations and 4DMR. Furthermore, ${\text {TMP}}_{\text {mean}}$ did not exceed 1.7 mmHg anywhere, well below the 5 mmHg observed in other aneurysmal patients.^42^ These effects, which all indicate low FL pressure, are likely due to the large number of communications (20) between TL and FL in this patient which act to minimise flow and pressure gradients between them. As a result, minor branch flow loss does not greatly affect reverse flow and ${\text {TMP}}_{\text {mean}}$ in this patient, but may do so in patients with fewer luminal communications. Even in this patient, the variations in TMP dynamics observed in the most rapidly growing region of the aorta suggest that minor branch flow loss may be a necessary modelling consideration in all patients for future predictive applications.

Low TAWSS and high OSI have also been linked with aneurysmal growth, both of which are observed throughout the thoracic FL of this patient.^20^ Minor branch flow loss progressively reduced TAWSS was along the aorta, up to 70% in the iliac branches. High TAWSS around luminal tears, such as at point β, has been suggested to influence their expansion,^11^ so a reduction of this magnitude may affect the prognostic value of simulations if they are incorporated into future clinical processes. In addition, OSI and ECAP were elevated with the inclusion of minor branches, with ECAP increasing by up to 75% at μ. Only $D_{\min }$ predicted values of ECAP over the suggested threshold of 1.4 Pa^-1^, which coincide with regions of calcification observed in the CTA data.

Both simulations achieved excellent qualitative agreement in velocity magnitude distributions with 4DMR. CFD predicted higher mean velocity than 4DMR at all planes, an effect commonly observed in comparisons between CFD and 4DMR.^12^ Minor branches progressively reduced the total plane flow rate and peak velocity in both lumens, leading to a favourable agreement with 4DMR overall. However, uncertainty in 4DMR velocity and flow rate may therefore be particularly high in this case (and other cases of AD) due to widespread regions of very low velocity and small luminal area compared to a healthy ascending aorta. We indeed observe greater discrepancies between CFD and 4DMR in the distal regions, though these were substantially reduced when minor branches were included.

Taking measurement errors into consideration, we cannot make firm conclusions on the relative accuracy of each CFD simulation using 4DMR data alone. However, we have reasonably assumed that minor branch flow loss is within the healthy physiological range in this patient due to the patency of the minor branches in CTA data and their lack of ischemic conditions. Based on these assumptions and the improved velocity agreement in $D_{\min }$, we suggest that a more accurate solution is obtained when minor branch flow loss is modelled. Looking beyond the accuracy of this specific case study, it is clear that minor branches affect the distribution of flow and pressure within the aorta to an extent that may be clinically relevant.

Various limitations and sources of simulation should be mentioned. 4DMR may underestimate flow rate and stroke volume by $\approx 30\%$ or more.^12,24^ However, a systematic offset in velocity does not affect our conclusions. Due to 4DMR imaging errors, the inlet velocity profile may exhibit weak velocity divergence, contravening the incompressibility assumption. In our simulations, the pressure coupling algorithm will locally redirect flow in the cells immediately neighbouring the inlet to preserve continuity. No issues in convergence and numerical stability were observed throughout this study, indicating that any such effects were minimal. Furthermore, any non-physiological impact will affect both cases equally and would not change our conclusions. Errors in target flow rate at the major outlets may also result from 4DMR uncertainties, and from using 4DMR data acquired two years later at the iliac branches. As equivalent boundary conditions were applied in each simulation, this also does not affect our conclusions. Although minor branch flow rates were consistent with literature values, simulated WSS and velocity distributions within them exceeded the physiological range. This either indicates insufficient branch diameter, or measurement errors in the Doppler ultrasound data.^23^ Furthermore, we have assumed constant minor branch diameter, but branches near the Adamkiewicz artery (vertebral level T11–T12) and vessels on the patient’s left side are typically larger and carry more flow.^23^ This again does not affect our general conclusions as we have only endeavoured to explore the global impact of minor branch flow loss on WSS distributions in the major branches and aortic lumina.

In terms of modelling assumptions, walls were assumed to be rigid. Compared with compliant simulations, the assumption of a rigid wall in TBAD has been shown to reduce peak TAWSS, expand regions of high oscillatory shear and low TAWSS, and affect the distribution of flow through luminal tears.^4,41^ However, the magnitude of these differences increased with flap compliance which is likely to be low due to the chronicity of this case of TBAD. Furthermore, time shifts in TL and FL pressure waves tend to zero in cases where large or numerous luminal communications are present; the assumption of a rigid flap has been previously justified on this basis.^7^ Even where flap compliance is large, TMP is minimally affected.^4,41^ Finally, we have used a RANS turbulence model for computational efficiency which may affect the accuracy of WSS distributions^1^; however, comparisons with equivalent laminar flow simulations (provided in Supplementary Material SM3) provided identical conclusions to those presented here.

This study represents an initial exploration of the impact of minor branches in a single case of TBAD (n=1) which may raise questions around the validity of our conclusions across a larger patient cohort. Population-wide anatomical variability of the minor branches is rather low. Variations in the location and shape of minor branches may occur due to spinal or congenital conditions, however these aspects are not likely to affect the broader conclusions of the current work which relate to bulk flow loss. We would expect that the naturally-occurring variation in net flow loss along the aorta amongst the patient population will affect the magnitude of observed haemodynamic differences when minor branches are modelled, an effect which may be compounded in the case of branch vessel occlusion. In AD patients, each minor aortic branch may originate from the TL or FL depending on the precise nature of the dissection. As we have seen, the relative number of patent branches originating from each lumen will affect the flow split and pressure dynamics between them. With suitable volumes of patient data, our proposed simulation technique may be deployed on a larger cohort of TBAD patients to explore these phenomena, offering novel insights into the involvement of branches in disease progression.

While the integration of simulation techniques into clinical workflows is outside the scope of this work, it is worth considering the implications and resource requirements in modelling minor aortic branches. CTA data, required to locate the minor branches, is routinely available in TBAD cases. Although 4DMR is not always included in routine TBAD imaging protocols, 4DMR-informed CFD is widely considered the gold-standard in aortic haemodynamic analysis and 4DMR acquisition is increasingly widespread.^27^ Reconstructing minor branches from CTA, tuning their outlet boundary conditions and the minor elevation in mesh size will marginally increase simulation times compared with other 4DMR-informed approaches. Further work may endeavour to account for minor branch flow loss without their explicit geometric inclusion to minimise resource requirements. The precise, clinically-permissible margins of error in haemodynamic data are not yet known in AD. If patient-specific individual minor branch flow rates are found to be needed for sufficient accuracy in future work, the acquisition of patient-specific Doppler ultrasound will require additional clinical resource. In future, modelling processes may be accelerated and streamlined *via* cooperation between Radiology departments, designated clinical modelling engineers, and with the use of automation tools for segmentation, boundary condition calibration and post-processing.

We have demonstrated that modelling minor branch flow loss reduces the magnitude of velocity, TMP and TAWSS to a considerable extent, particularly in the abdominal and iliac regions. Minor branch inclusion also affects the intra-luminal pressure dynamics in the proximal aorta, where substantial FL dilatation was observed in this patient. OSI and ECAP are elevated when minor branches are included, both in the thoracic FL, where aneurysmal development is observed longitudinally across both sets of 4DMR data and in the abdominal TL, where calcification is observed in CTA data. Because these differences are observed in quantities of potential clinical significance, accounting for minor branch flow loss may be an essential consideration as simulation techniques progress closer to clinical application. Including minor branches in simulations may contribute to our understanding of post-intervention complications, including the patency of intercostal grafts, and may eventually assist in planning interventions for TBAD.

## Supplementary Information

Below is the link to the electronic supplementary material.

(PDF 8677 kb)
