# Supplementary material for: The Influence of Minor Aortic Branches in Patient-Specific Flow Simulations of Type-B Aortic Dissection
Source: Ann Biomed Eng. 2023 Mar 26;51(7):1627–44. doi: 10.1007/s10439-023-03175-4 (PMC10264290; doi:10.1007/s10439-023-03175-4)
Supplement: Supplementary file 1 — (PDF 8677 kb) [file 10439_2023_3175_MOESM1_ESM.pdf]

Supplementary Material

The influence of minor aortic branches in patient-specific simulations of Type-B Aortic Dissection

SM1 Segmentation & registration

Using Simpleware ScanIP (Synopsys Inc., CA, USA), an initial mask of the aortic volume was manually segmented from CTA data to include the aortic root, the major aortic branches, and the visible extent of all segmental arteries, which we will call M0. Because the segmental arteries are small compared to CTA resolution, their surfaces are rough, noisy, and unsuitable for direct use in CFD simulations, as shown in Fig. 1 (in the main text), and Fig. Ia below. At this stage, the locations of intimal tears were confirmed with clinicians. A copy of this mask (M1) was created in which the segmental branches were removed, leaving a small hole at the location of their bifurcations, as shown in Fig. Ib. The surface of this mask was smoothed using Meshmixer (Autodesk Inc., CA, USA) while retaining the holes (M2).

To register the CTA domain onto the 4DMR domain, a mask of the 4DMR domain was manually extracted using 4DMR magnitude images in ScanIP (M3), as shown in grey in Fig. I. This mask includes only the major geometric features of the aorta due to the relatively low resolution of 4DMR. Next, non-rigid landmark-based registration of the smoothed CTA mask (M2) onto the 4DMR mask (M3) was performed in MATLAB (Mathworks, Natick, MA, USA) using non-rigid Continuous Point Drift (CPD) registration[SM1] to produce M4. Tears in M4 were then manually readjusted to ensure their shape and size remained faithful to the CTA data (M5). Final smoothing in Meshmixer resulted in M6, which retains the small holes at the bifurcation points so that the reconstructed minor branches could be reattached in the correct location. A copy of M6 with these holes carefully smoothed flat, M7, is the final CFD domain without segmental branches and is shown in Fig. I.

Minor branches were omitted from the preceding steps as they became excessively deformed during the non-rigid registration process. Instead, the centrelines of all minor branches were extracted from M0 using ScanIP. Using ANSYS SpaceClaim (ANSYS Inc., PA, USA), they were reconstructed as cylinders with a constant 1.5mm diameter to approximately match the mean diameter observed in the CTA data and results from anatomical studies[SM2,SM3]. In reality, the segmental diameters will vary along the length of the aorta, however as we are primarily interested in the global impact of minor branch outflow rather than the haemodynamics within them, this assumption suffices. Each branch was swept along a length of 15mm (10 diameters) from the bifurcation point to ensure their outlets did not impact flow in the aorta[SM4]. Importing these reconstructed vessels into ScanIP along with M6, each branch was moved to its appropriate bifurcation point and united with the

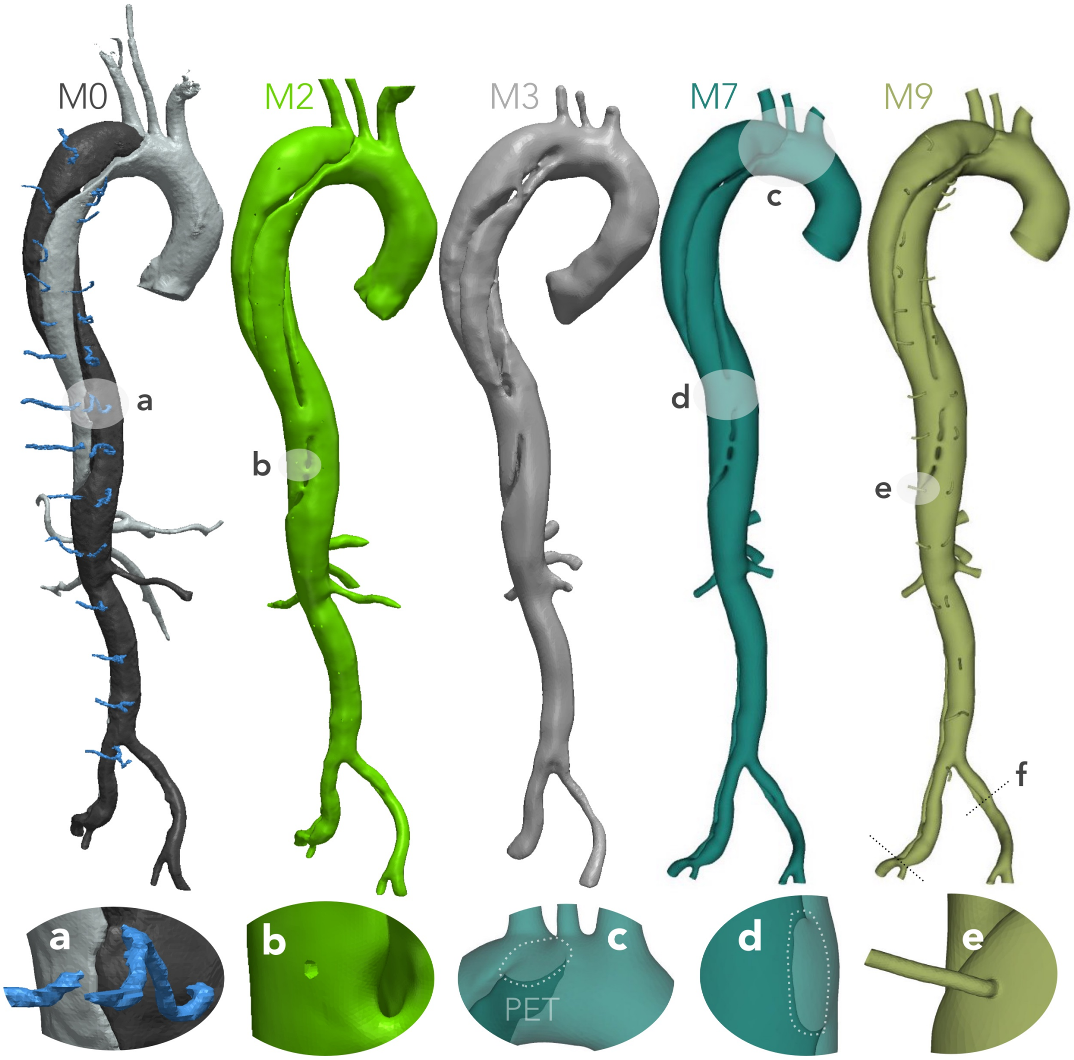

Figure I: *Images of the aortic geometry at sequential stages of the segmentation and registration process, including the final geometries for D and* D_{min}*: M7 and M9. Detail views are the baseline minor branches before reconstruction (a), the 'holes' marking the locations of removed minor branches (b), the primary entry tear (PET) (c), the first re-entry tear (d) and a reconstructed minor branch showing its smoothed bifurcation (e). Images of M1, M4-M6 and M8 can be seen in Fig. II.*

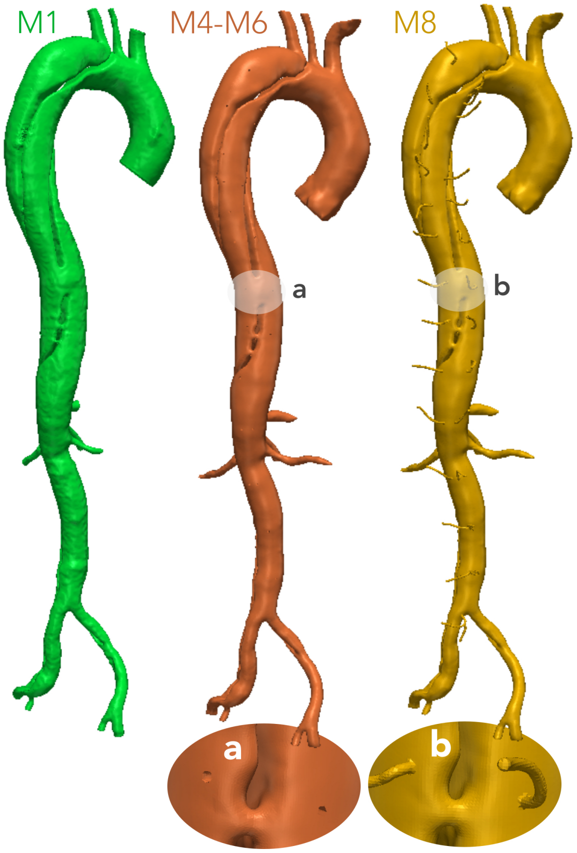

Figure II: *Images of the aortic geometry at sequantial stages of the segmentation and registration process, M1, M4-M6, and M8. M4-M6 look identical externally, hence their shared image. detail (a) and (b) show the respective locations of holes and reconstructed minor branches. The remaining masks are shown in Fig. I*

aortic mask to produce M8. As M8 includes sharp edges at each bifurcation, which is not physiologically accurate[SM5], the edges were rounded in Meshmixer to produce the final domain with segmental branches (M9), shown in Fig. I. We will refer to M7 and M9, the cases without and with minor branches, as D and D_{min}, respectively. Both domains include all major branches and are physically identical with exception to the minor branches. Images of masks M1, M4-M6, and M8 are shown in Fig. II. M4-M6 are grouped as only minor changes were made to the PET to align with the CTA data after registration, so masks looked externally identical.

SM2 Mesh Independence

To determine an appropriate mesh resolution for the simulations in this study, a number of relevant haemodynamic quantities were evaluated on three successively refined meshes using

percentage changes and Grid Convergence Index (GCI)[SM6,SM7]. In each mesh, generated with Fluent Mesh (ANSYS Inc., PA, USA), tetrahedral elements were sized with sensitivity to proximity and curvature, a growth rate of 1.2, and maximum/minimum cell sizes as indicated in Tables II and I. Ten near-wall (inflation) layers were used in each mesh, as recommended when using the k-ω SST model. A first cell height of 0.05mm was applied in all meshes, aiming for a y+≈1 to ensure that the first cell lay within the viscous sublayer. This value resulted in a mean y+ of 0.823 across all walls, and a maximum of 3.730 at peak systole, falling well within the recommended mean value of 5. GCI was calculated as a percentage using the following equations, where c, m and f correspond to quantities from the coarse, medium and fine meshes, respectively:

$$r_{f,m} = \left(\frac{N_f}{N_m}\right)^{\frac{1}{3}} \approx r_{m,c} = \left(\frac{N_f}{N_m}\right)^{\frac{1}{3}} \tag{S1}$$

$$r = \frac{r_{f,m} + r_{m,c}}{2} \tag{S2}$$

$$p = \frac{ln\left(\frac{|f_c - f_m|}{|f_m - f_f|}\right)}{ln(r)} \tag{S3}$$

$$E_{f,m} = \frac{\left(\frac{|f_m - f_f|}{f_f}\right)}{r^p - 1} \qquad E_{m,c} = \frac{\left(\frac{|f_c - f_m|}{f_m}\right)}{r^p - 1} \tag{S4}$$

$$GCI_{f,m} = F_S|E_{f,m}| \qquad GCI_{m,c} = F_S|E_{m,c}| \tag{S5}$$

N is the number of elements in the mesh, f is the examined variable of interest, and F_S is a safety factor of 1.25[SM7,SM8]. By halving the minimum and maximum element sizes with each successive refinement, a refinement factor of $r = 2$ was applied, exceeding the minimum recommended factor of 1.33[SM6].

The minor branches, and thus their mesh sizing requirements, were considerably smaller than the rest of the geometric features of the domain. As such, appropriately assessing their resolution requirements in the context of a global mesh refinement study would be prohibitively expensive with our computational resources. To address this issue, a preliminary refinement study was first performed on a single-branch segment of the wall (shown in Fig. III c), the resolution of which would later be fixed across the full mesh refinement study whilst the global mesh sizes were adjusted. As the minor branch sizing would be equivalent or smaller than most refined global mesh, this was deemed a suitable compromise for computational efficiency.

In the single-branch refinement study, a scaled-down equivalent of the aortic inlet flow waveform was applied at the inlet with a total flow rate over the cycle equal to the maximum branch flow measured by Koyanagi et. al[SM3]. A constant pressure outlet equal to the diastolic pressure target (P_D) was applied at the end of the minor branch.

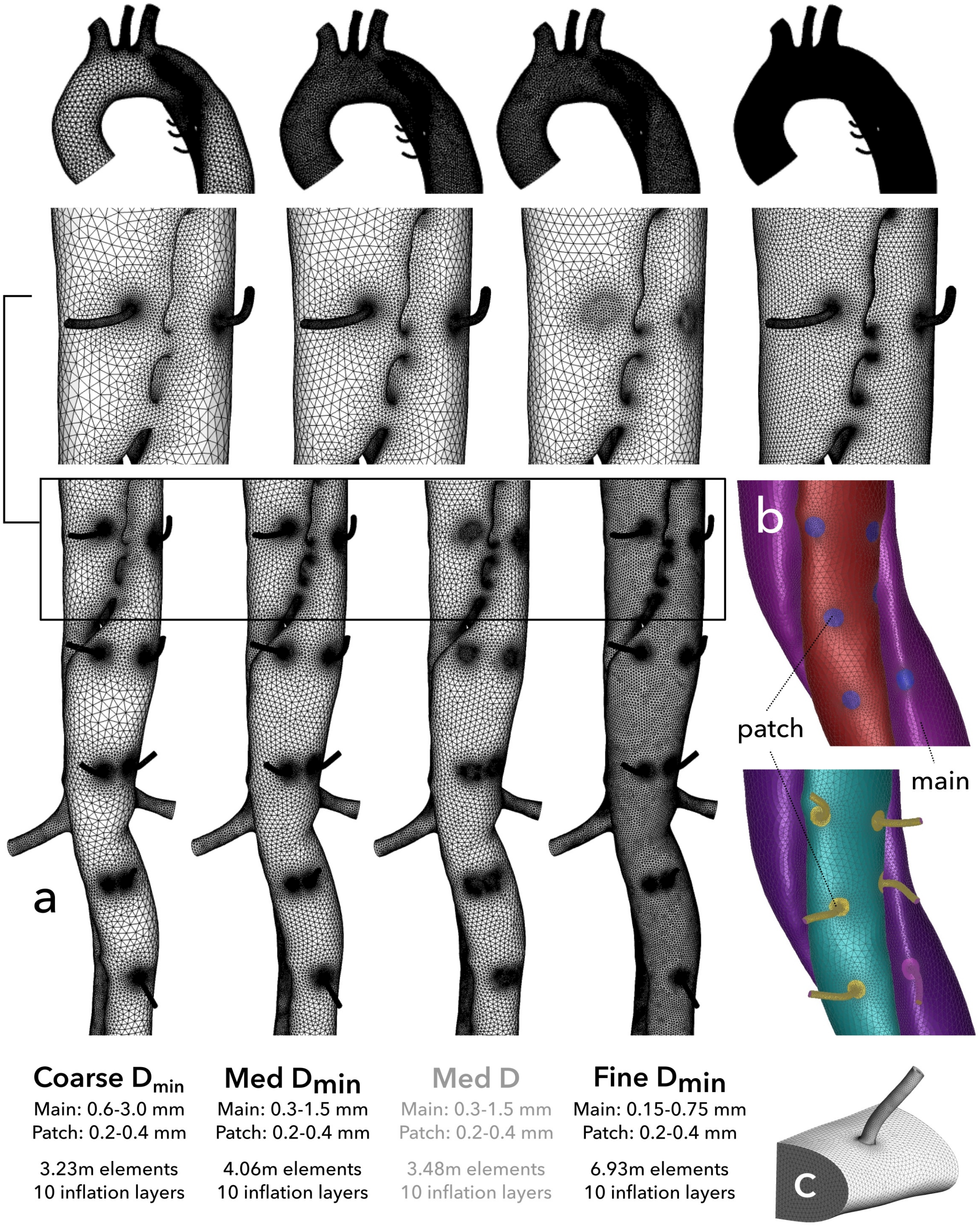

Figure III: *Images of the final mesh for D and D_{min} with mesh details indicated below (a), the minor branch mesh 'patches' (b), and the geometric domain used for the single-branch mesh independence study (c)*

Metric	*fine*	*medium*	*coarse*	$\%_{f,m}$	$\%_{m,c}$	$GCI_{f,m}$	$GCI_{m,c}$
$cell_{min}$ *(mm)*	0.05	0.1	0.2	100	100		
$cell_{max}$ *(mm)*	0.1	0.2	0.4	100	100		
Elements	300448	111188	54718	-63	-51		
Inlet P_{max}	123.59	124.24	125.9	-0.52	**-1.32**	0.543	**1.075**
Outlet v_{max}	3.314	3.312	3.348	0.06	**-1.08**	-0.545	**0.080**
Domain v_{max}	3.314	3.33	3.38	-0.48	**-1.48**	1.363	**0.883**

Table I: *Mesh properties, haemodynamic quantities of interest, and GCI values for the single-branch segment.*

Maximum inlet pressure, maximum velocity at the branch outlet, and maximum velocity throughout the volume were compared, and shown in Table I. As we did not closely assess the precise flow distribution within the minor branches in this study, simply the impact of their flow loss, WSS indices were not considered in the single-segment study. Pressure and velocity metrics changed by $< 2\%$ and GCI indices were below 1.1% between coarse and medium meshes, so the coarse parameters of 0.2-0.4mm were concluded to be appropriate to resolve the minor branches up to their maximal flow output.

To mesh the full domain, the minor branches in D_{min} or their equivalent location in D, were separated from the surrounding walls as a 'patch' where the previously determined minor branch element size range of 0.2-0.4mm was applied, as shown in Fig. IIIb. In doing so, meshes D and D_{min} would be refined identically in the branch locations while the global mesh refinement could be adjusted in isolation, as evidenced in Fig. IIIa. The full mesh refinement study was carried out on the D_{min} domain, where we compared systolic and diastolic pressure at the inlet, maximum velocity in the full volume at peak systole, maximum WSS magnitude on the wall at peak systole, mean OSI, mean and maximum TAWSS, and mean velocity on a proximal and a distal plane (a and g), per lumen, at peak systole. Between fine and medium meshes, the maximum difference in any quantity was $< 5\%$ while the maximum difference between medium and coarse meshes was 28%, so the medium mesh resolution was selected for the final study. Across all meshes, GCI did not exceed 6.5% for any quantity in any mesh.

Furthermore, to ensure that any differences between fine and medium meshes would not affect the conclusions of this work, WSS distributions were assessed qualitatively. TAWSS, OSI and ECAP distributions from medium and fine meshes are shown in Fig. IV. The lack of notable qualitative differences further supports the use of the medium mesh in this study.

Metric	*fine*	*medium*	*coarse*	$\%_{f,m}$	$\%_{m,c}$	$GCI_{f,m}$	$GCI_{m,c}$
global $cell_{min}$ *(mm)*	0.15	0.30	0.60	100	100		
global $cell_{max}$ *(mm)*	0.75	1.50	3.00	100	100		
refinement factor (r)	2	2					
inflation layers	10	10	10				
first cell height (mm)	0.05	0.05	0.05				
Elements	6930146	4055878	3231971	-41.47	-20.31		
systolic CFL_{max}	82.5	65.0	71.9	-21.09	10.54		
systolic CFL_{mean}	4.3	5.0	5.3	18.97	4.33		
P_S	127.9	128.2	128.8	0.21	0.48	**0.210**	0.472
P_D	82.6	82.9	82.9	0.36	0.11	**0.649**	0.194
v_{max}	2.582	2.558	2.617	-0.93	2.27	**0.809**	1.986
τ_{max}	161.35	153.55	154.00	-4.83	0.29	**6.412**	0.387
OSI_{mean}	0.1598	0.1558	0.1115	-2.47	-28.50	**0.300**	3.469
$TAWSS_{max}$	96.202	93.260	93.838	-3.06	0.62	**4.758**	0.965
$TAWSS_{mean}$	2.346	2.309	2.869	-1.61	24.27	**0.145**	2.193
$\overline{v}_{sys}$ *a, TL*	0.410	0.407	0.408	-0.79	0.25	**1.441**	0.461
$\overline{v}_{sys}$ *g, TL*	0.848	0.848	0.851	-0.03	0.44	**0.002**	0.035
$\overline{v}_{sys}$ *a, FL*	0.363	0.370	0.374	1.95	1.03	**5.256**	2.770
$\overline{v}_{sys}$ *g, FL*	0.410	0.389	0.389	-0.06	-0.07	**0.420**	0.491

Table II: *Mesh properties, haemodynamic quantities of interest, and GCI values from the* D_{min} *refinement study using a uniform 0.2-0.4mm branch patch resolution. Each mesh is shown in Fig. III*

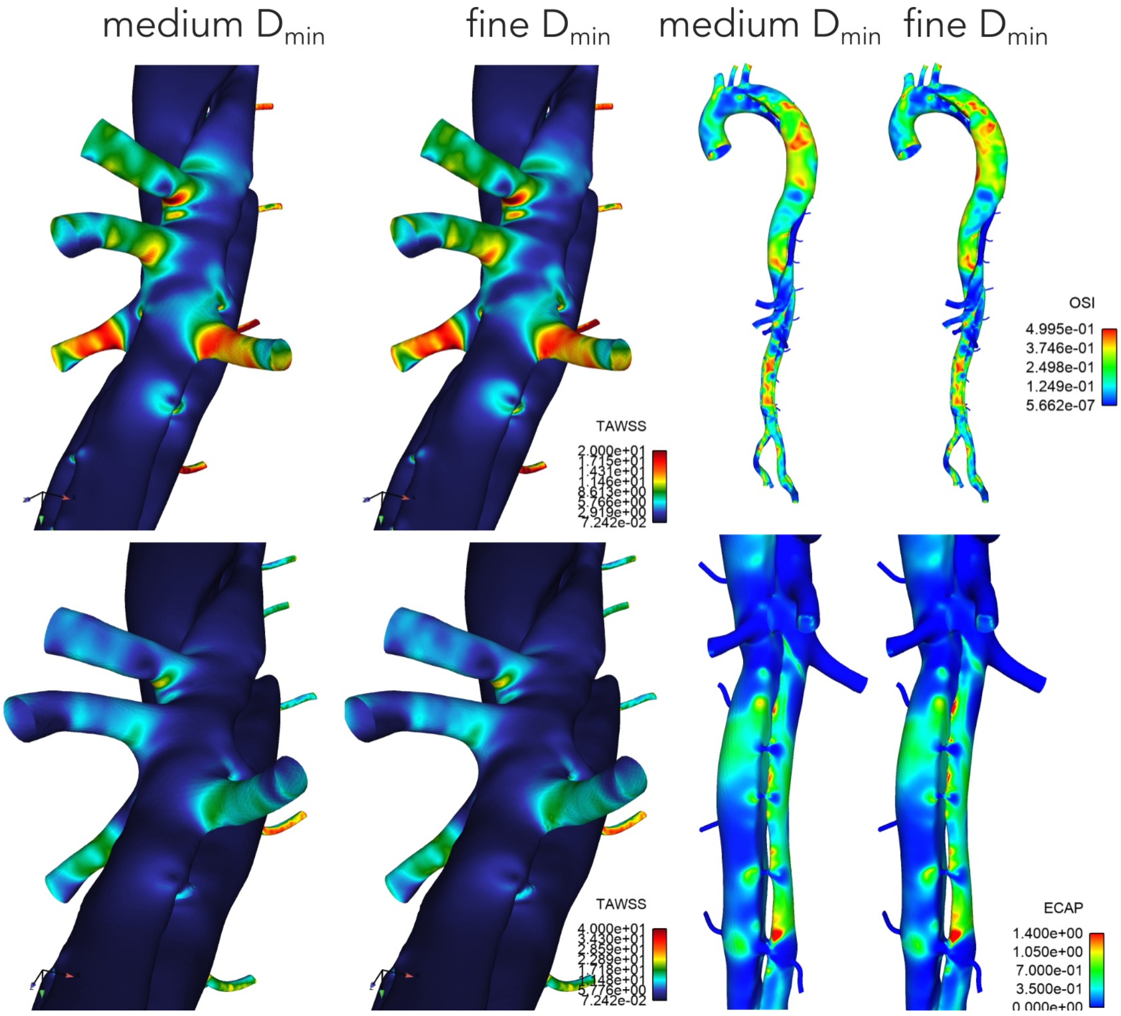

Figure IV: *Detail views of the TAWSS, OSI and ECAP distributions from the medium and fine D_{min} meshes.*

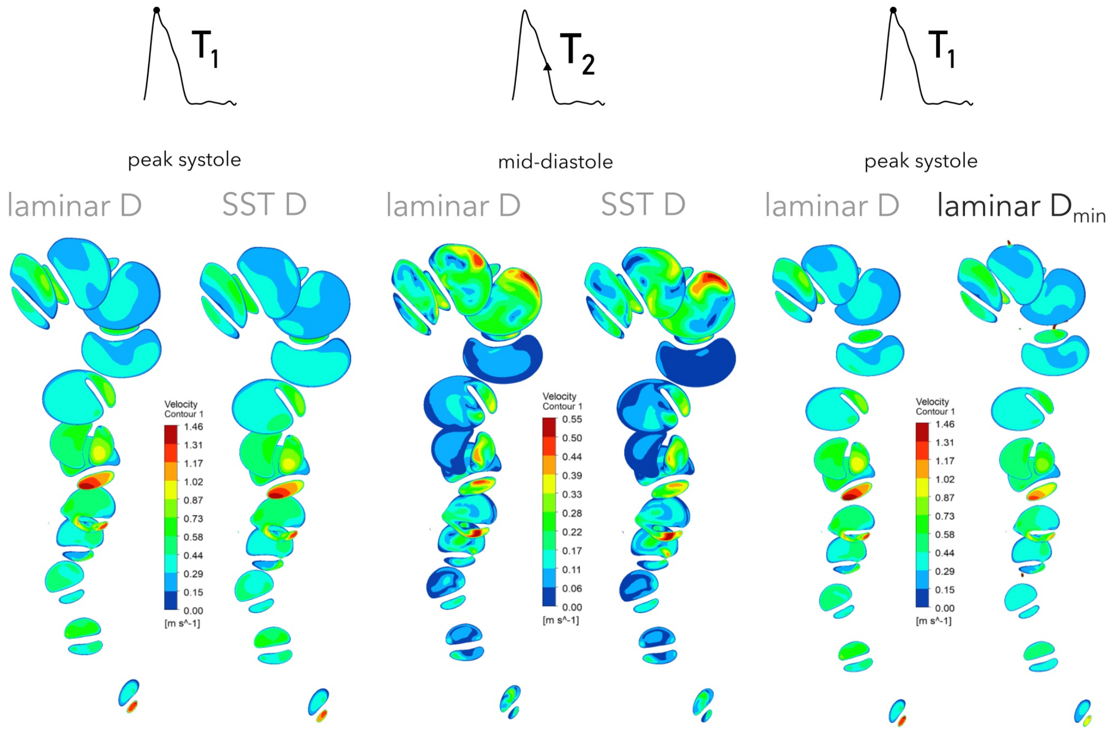

Figure V: *Velocity contours at peak systole (T_1) and mid-diastole (T_2) from laminar and turbulent simulations of D (a and b), and a comparison between laminar simulations of D and D_{min} at T_1 (c).*

SM3 Comparing Laminar and RANS Simulations

Using the medium D and D_{min} meshes, laminar and turbulent simulations were compared to assess whether the choice of turbulence model would impact the conclusions of this study. Velocity contours from the respective laminar and turbulent simulations of D are shown at peak-systole and mid-diastole in Fig. V. A comparison between laminar D and D_{min} simulations are also shown at peak systole. The use of a turbulence model results in a slight reduction in peak velocity and some minor differences in the low velocity distribution within the FL, particularly in the region immediately distal to the primary entry tear. Regions of high velocity were minimally affected. These trends were observed also in D_{min}. The marked reduction in velocity along the aorta with the inclusion of minor branches occurred regardless of turbulence model, therefore, study conclusions on velocity and agreement with 4DMR hold.

To ensure that any differences in velocity did not affect WSS metrics to an extent that would affect the study conclusions, qualitative TAWSS, OSI and ECAP distributions were compared between laminar and k-ω SST simulations of D, as shown in Fig. VI. Furthermore, the quantitative differences in TAWSS and ECAP are also provided for laminar and turbulent

		**D**		D_{min}		$\frac{\|D-D_{min}\|}{D_{min}}$ (%)	
		Laminar	SST	Laminar	SST	Laminar	SST
TAWSS (Pa)	δ	24.90	22.60	25.40	23.60	2.0	4.2
	β	17.40	15.40	10.50	11.40	65.7	35.1
	γ	31.50	26.50	21.70	17.20	45.2	54.1
	η	21.10	17.20	13.10	10.10	61.1	70.3
ECAP (Pa^{-1})	σ	0.87	1.07	1.07	1.23	18.7	13.0
	ϵ	1.15	1.08	1.60	1.61	28.1	32.9
	μ	0.80	0.95	1.49	1.66	46.3	42.8

Table III: *Values of TAWSS and ECAP from laminar and turbulent simulations at the points indicated in Figs. 5 and 6 in the main text. The percentage difference between D and D_{min} values are provided for both laminar and turbulent cases, indicating the same trends.*

simulations of D and D_{min} at points β, δ, γ, ν, σ, ϵ and μ in Table III (point locations indicated on Figs. 5 and 6 in the main text). While higher TAWSS and lower ECAP values are observed in the laminar case, the percentage differences observed between D and D_{min} are of a similar magnitude. As such, the choice of turbulence modelling assumption does not affect the conclusions of this study.

While distributions of TAWSS, OSI and ECAP are qualitatively similar, TAWSS reached higher levels in the laminar simulation while ECAP was lower. However, as shown in Table, trends between D and D_{min} remained the same with similar percentage differences in both laminar and turbulent simulations. Therefore, study conclusions surrounding WSS indices are not affected by the use of a turbulence model. Due to aforementioned differences in the velocity beyond the primary entry tear, OSI distributions were slightly altered in the proximal FL, but also not to a degree that affects our conclusions.

Finally, Fig. VII shows TAWSS, OSI and ECAP contours from laminar simulations of D and D_{min}, identical to the turbulent versions in the main text, indicating that the same trends are observed, namely a progressive reduction in TAWSS and an increase in ECAP when minor branches are included. This confirms that the use of the k-ω SST turbulence model has not affected the conclusions of this study.

SM4 Derivation of minor branch flow rates

Because the minor branches including the segmental arteries and IMA are smaller than the resolution of 4DMR, their individual mean flows cannot be extracted. Any mean flow loss between aortic planes where major branches were not present could be attributed to minor

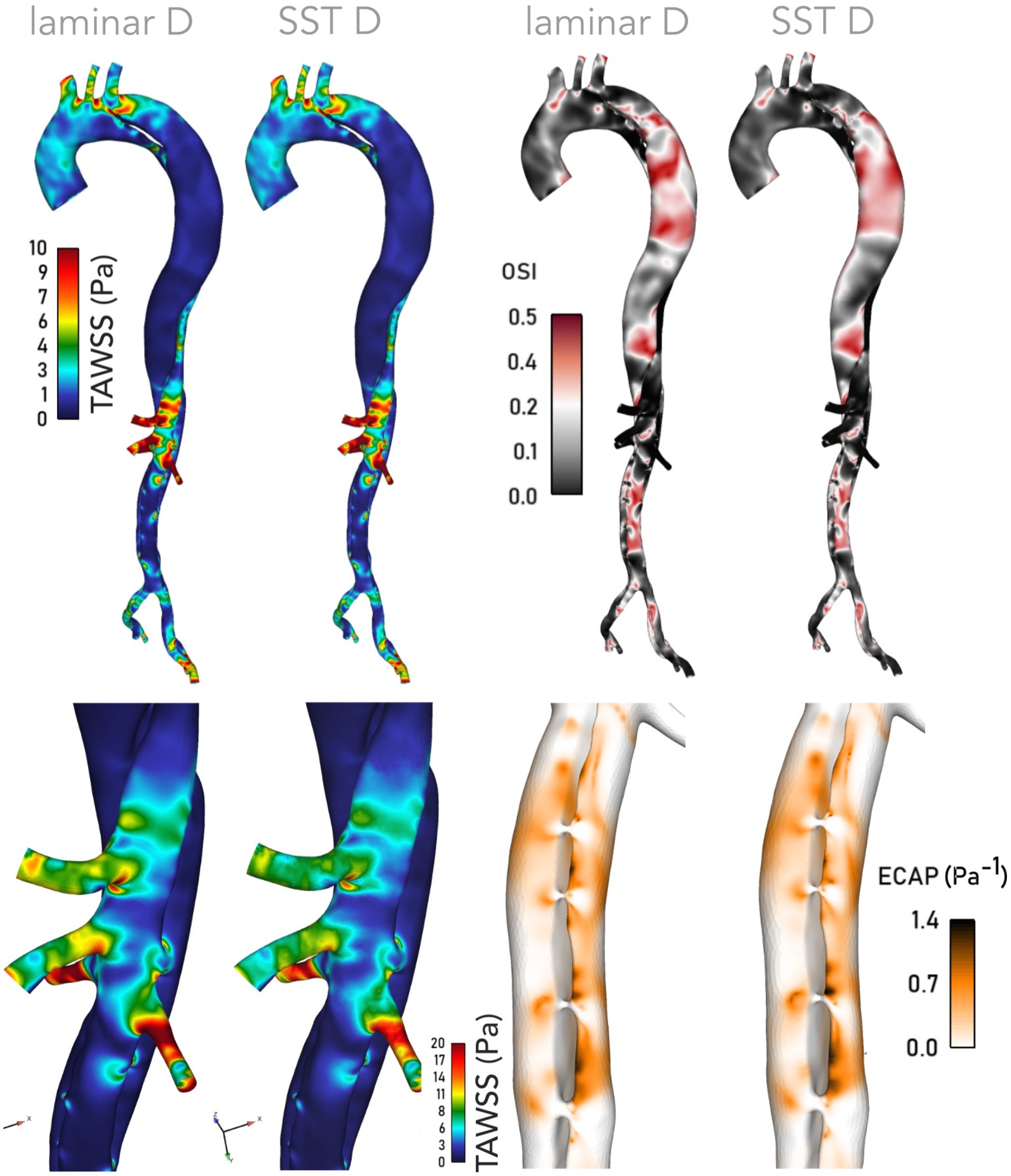

Figure VI: *A qualitative comparison of TAWSS, OSI and ECAP distributions from laminar and $k-\omega$ SST simulations of D, indicating increased TAWSS and reduced ECAP values in the laminar simulations, but otherwise near-identical qualitative distributions.*

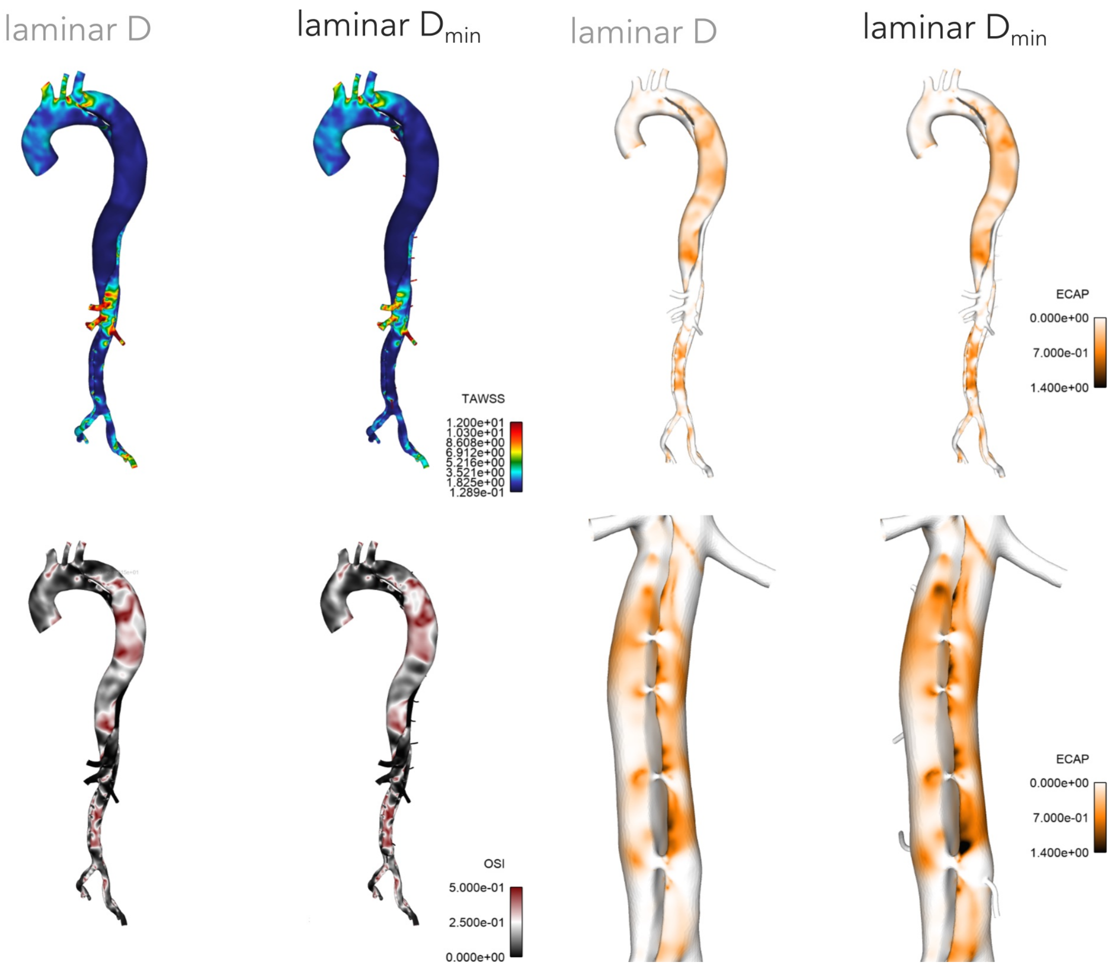

Figure VII: *A qualitative global comparison of TAWSS, OSI and ECAP distributions in laminar simulations of D and* D_{min}*, showing identical trends to the turbulent case comparison.*

branch outflow, however, mass is not conserved in 4DMR and an uncertainty in flow rate measurements is of the order of the total flow loss through all intercostal arteries. Furthermore, such measurements do not provide information on how to split the flow appropriately between S1 and S2, or S4 and the IMA as both sets of branches lie between the same two measurement planes and it is known that flow from the left segmental arteries are generally higher than the right. This approach also cannot be applied at SC where only one pair of branches exist at the same level as the major abdominal arteries. Instead, we refer to Koyanagi et. al.[SM3] and Erden et. al[SM9], who measured segmental and IMA velocities, respectively, in healthy subjects using Doppler ultrasound. The mean flow loss for each individual branch from these studies was used to assign the target flow that would leave each group of minor branches. The resulting supra-aortic, abdominal, and segmental flow splits of 26%, 40% and 20% in D_{min} align with previously reported flow splits.

As all minor branches arise distal to the LSA, supra-aortic flow targets were identically set in cases D and D_{min}. In D, the total flow loss from S1-S3 was added to the CT, SMA and renal arteries proportionally based on the ratio of their mean flows in D_{min} to the branch group flow. For example at the RRA:

$$\bar{Q}^{D}_{RRA} = \bar{Q}^{D_{min}}_{RRA} \left(1 + \frac{\bar{Q}_{S1} + \bar{Q}_{S2} + \bar{Q}_{S3}}{\sum \bar{Q}^{C*}_{abdominal}}\right) \tag{S6}$$

where superscripts D and D_{min} correspond to mean flow targets in cases D and D_{min}, respectively, and the summed quantities refer to the total abdominal group flow in D_{min}. Flow loss from S4 and the IMA was similarly distributed between the iliac outlets, for example at REI:

$$\bar{Q}^{D}_{REI} = \bar{Q}^{D_{min}}_{REI} \left(1 + \frac{\bar{Q}_{S4} + \bar{Q}_{IMA}}{\sum \bar{Q}^{C_{min}}_{iliac}}\right) \tag{S7}$$

SM5 Velocity contours & Bland-Altman plots

Velocity contours are shown at T_1 and T_2 on all planes not included in the main text in Fig. VIII, while all Bland-Altman plots from T_1 and T_2 are shown for reference in Figs. IX and X.

SM6 Error quantification

On a given analysis plane and time point (T_1 or T_2), the mean difference in pointwise velocity was evaluated as a percentage of the mean inlet velocity over all n points on the plane:

$$\text{mean error} = \frac{1}{n} \sum_{k=1}^{n} \frac{v_k^{CFD} - v_k^{4DMR}}{\bar{v}_{inlet}} \tag{S8}$$

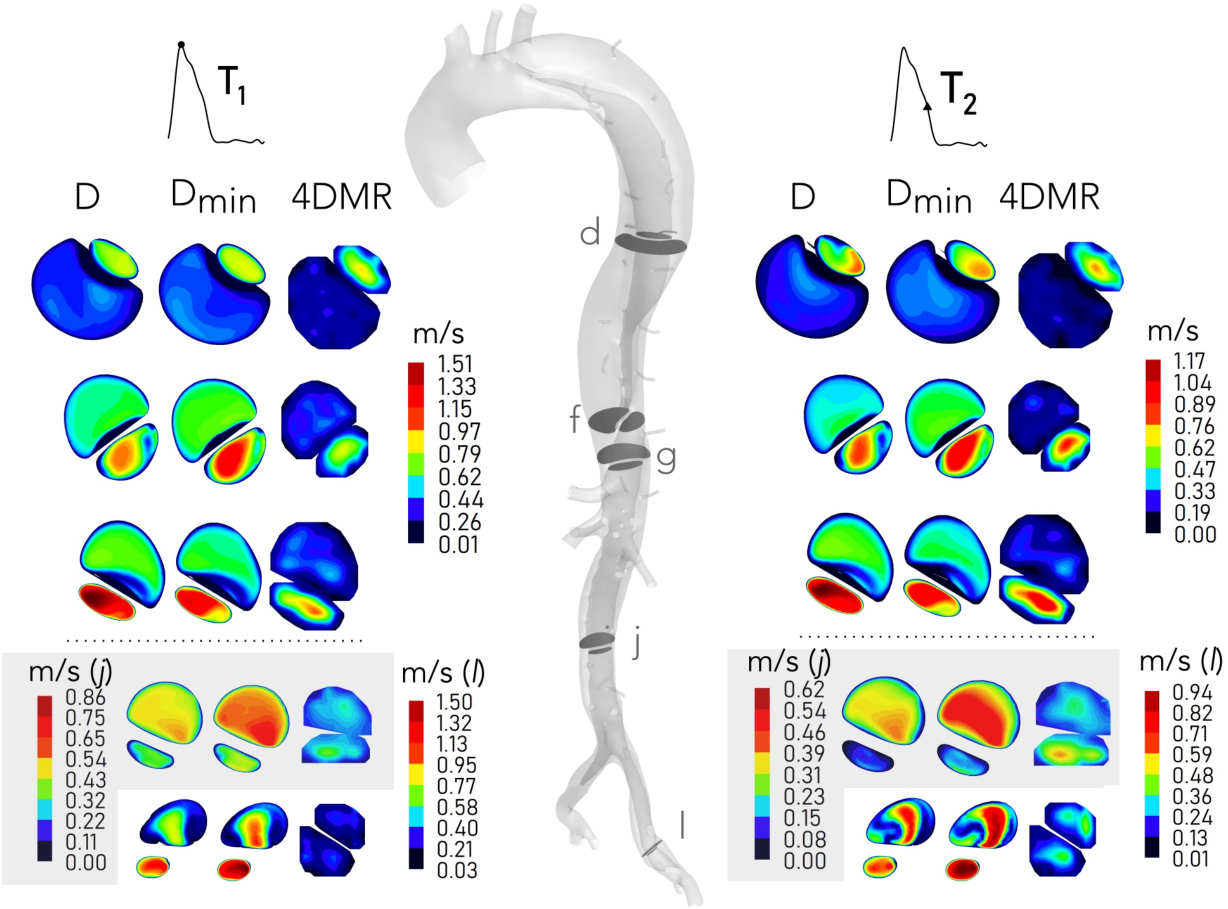

Figure VIII: *A comparison of velocity magnitude contours between CFD cases and 4DMR at peak systole and mid-diastole,* T_1 *and* T_2*, on all analysis planes omitted from the main text.*

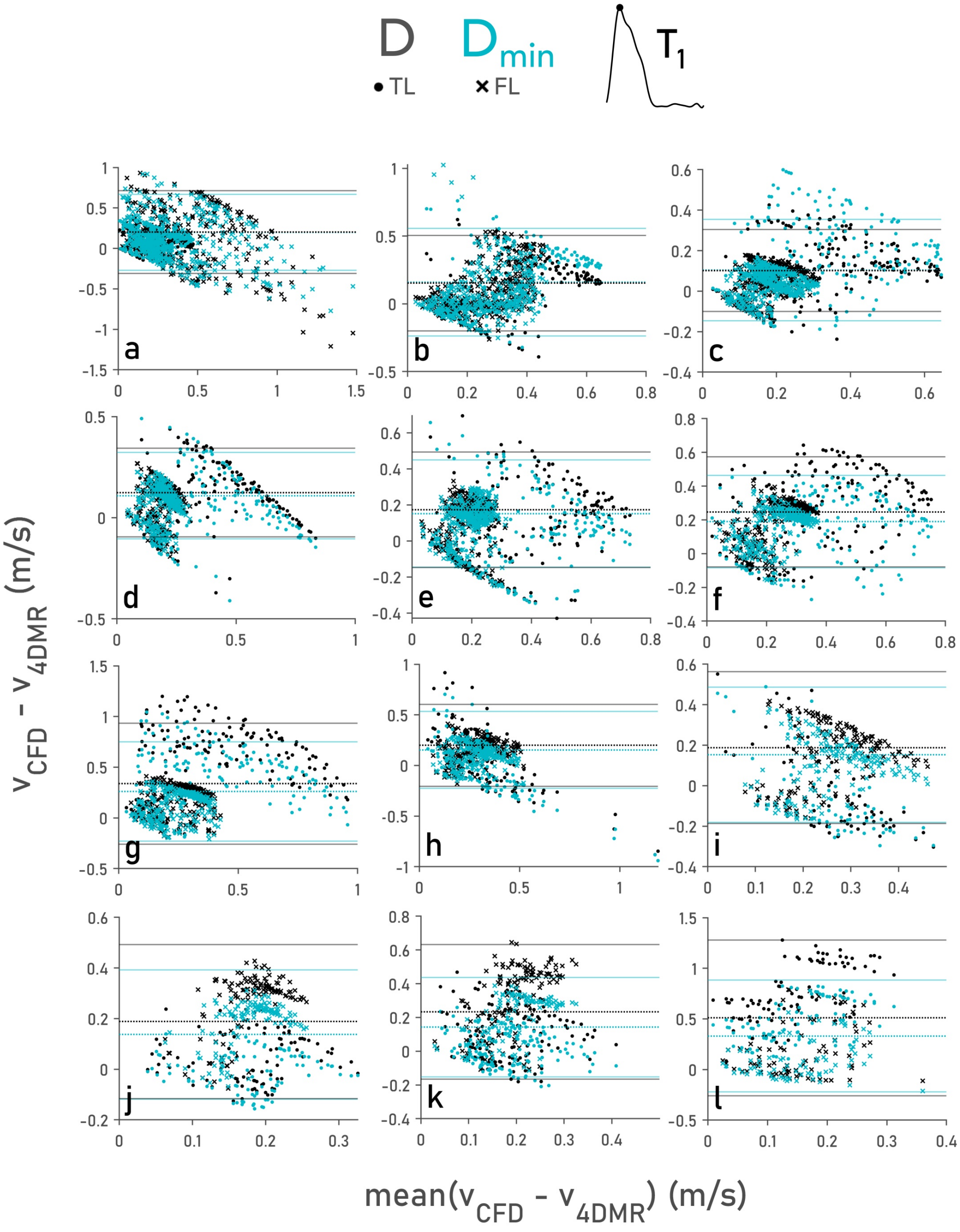

Figure IX: *Bland-Altman plots comparing simulated and 4DMR velocity magnitude from D and D_{min} at peak systole (T_1). Analysis planes are shown schematically in Fig. 3 in the main text. Bias and limits of agreement are shown as dotted and solid lines, respectively, for each case.*

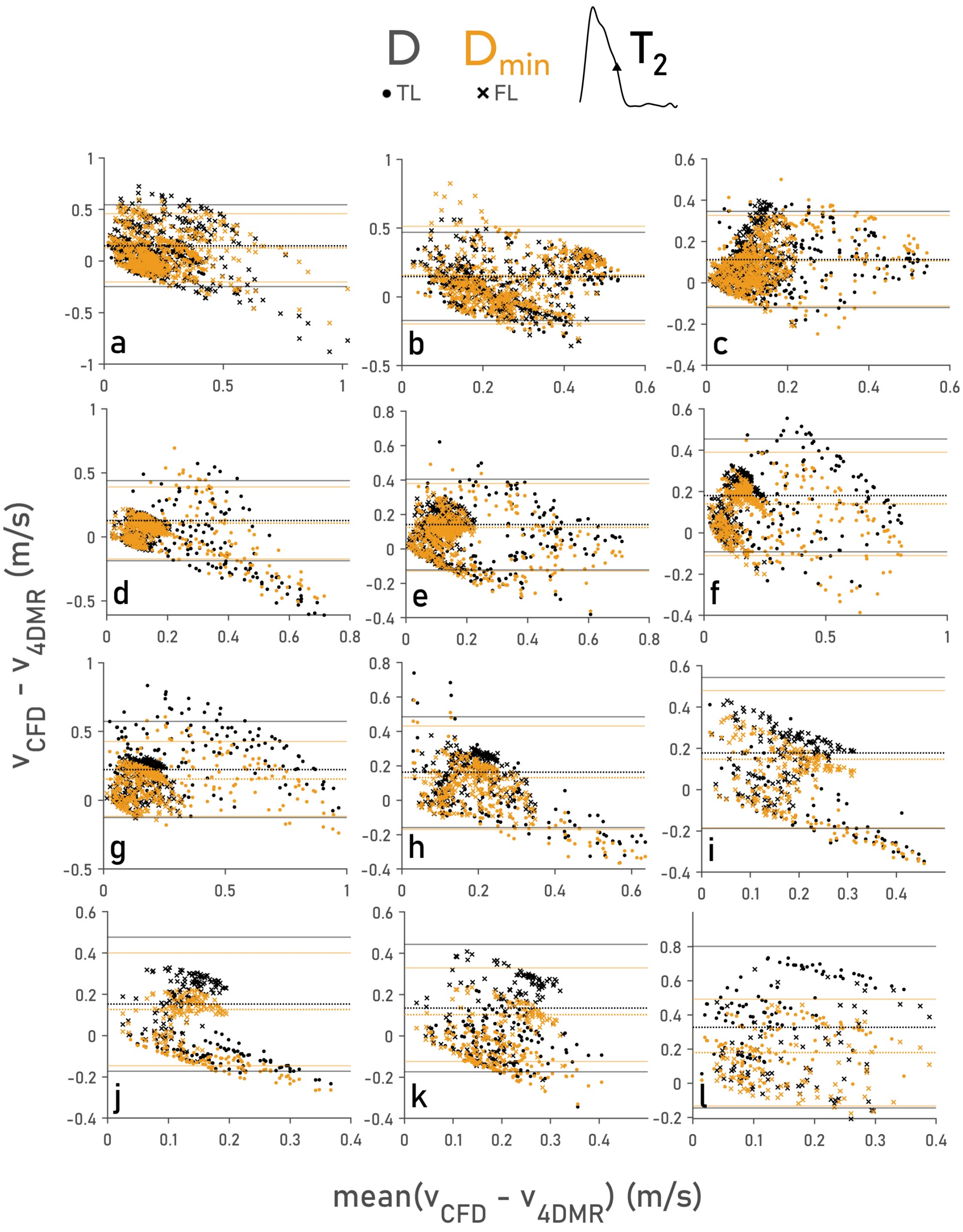

Figure X: *Bland-Altman plots comparing simulated and 4DMR velocity magnitude from D and D_{min} at peak systole (T_2). Analysis planes are shown schematically in Fig. 3 in the main text. Bias and limits of agreement are shown as dotted and solid lines, respectively, for each case.*

	**TL (%)**		**FL (%)**	
plane	D	D_{min}	D	D_{min}
a	24.3	28.3	53.8	43.9
b	42.6	58.5	47.3	46.0
c	38.6	33.6	48.2	27.4
d	68.1	62.8	28.9	23.0
e	46.0	45.7	43.5	35.8
f	55.4	38.6	56.9	46.0
g	103.9	61.3	51.0	41.1
h	48.2	51.0	51.6	36.4
i	54.7	55.7	55.7	41.7
j	29.9	40.4	60.6	38.6
k	29.2	33.0	56.6	30.8
l	135.6	68.7	62.5	40.4
average	56.4	48.1	51.4	37.6

Table IV: *Mean percentage errors (Equation S8) in velocity magnitude between each case (D and D_{min}) and 4DMR, relative to the mean inlet velocity at T_2. Planes $a - l$ are shown schematically in Fig. 3 in the main text.*

To calculate $v_k^{CFD} - v_k^{4DMR}$, CFD velocity magnitude was interpolated onto the 4DMR grid points on each plane. In this way, the error quantifies differences in spatial velocity distribution.

The error in peak velocity within each lumen on a given plane λ was evaluated at peak systole (T_1) using the maximum values of velocity on each plane relative to the maximum inlet velocity:

$$\text{peak error} = \frac{max(v_\lambda^{CFD}) - max(v_\lambda^{4DMR})}{max(v_{inlet})} \tag{S9}$$

Mean and peak velocity errors (Equation S8 and S9), are shown at peak systole (T_1) in Table 3 in the main text, and mean errors are provided at mid-diastole (T_2) in Table IV.

References

[SM1] Hu, Y., Rijkhorst, E., Manber, R., Hawkes, D. & Barratt, D. Deformable Vessel-Based Registration Using Landmark-Guided Coherent Point Drift. *Medical Imaging And Augmented Reality.* pp. 60-69 (2010)

[SM2] Son, J., Smedts, F., Korving, J., Guyt, A. & Kok, L. Intercostal artery: histomorphometric study to assess its suitability as a coronary bypass graft. *Ann. Thorac. Surg.*. **56**, 1078-1081 (1993,11)

[SM3] Koyanagi, T., Kawaharada, N., Kurimoto, Y., Ito, T., Baba, T., Nakamura, M., Watanebe, A. & Higami, T. Examination of intercostal arteries with transthoracic Doppler sonography. *Echocardiography.* **27**, 17-20 (2010)

[SM4] Madhavan, S. & Kemmerling, E. The effect of inlet and outlet boundary conditions in image-based CFD modeling of aortic flow. *Biomed. Eng. Online.* **17**, 66 (2018,5)

[SM5] Kazakidi, A., Plata, A., Sherwin, S. & Weinberg, P. Effect of reverse flow on the pattern of wall shear stress near arterial branches. *J. R. Soc. Interface.* **8**, 1594-1603 (2011,11)

[SM6] Celik, I., Ghia, U., Roache, P., Freitas, C. & Raad, P. Procedure of Estimation and Reporting of Uncertainty Due to Discretization in CFD Applications. *J. Fluids Eng.*. **130**, 078001 (2008,7)

[SM7] Craven, B., Paterson, E., Settles, G. & Lawson, M. Development and verification of a high-fidelity computational fluid dynamics model of canine nasal airflow. *J. Biomech. Eng.*. **131**, 091002 (2009)

[SM8] Armour, C., Guo, B., Pirola, S., Saitta, S., Liu, Y., Dong, Z. & Xu, X. The influence of inlet velocity profile on predicted flow in type B aortic dissection. *Biomech. Model. Mechanobiol.*. **20**, 481-490 (2021)

[SM9] Erden, A., Yurdakul, M. & Cumhur, T. Doppler waveforms of the normal and collateralized inferior mesenteric artery. *AJR Am. J. Roentgenol.*. **171**, 619-627 (1998)
